# In-Hospital Mortality Predictors and a Bayesian Weighted-Incidence Antibiogram in Infective Endocarditis: A Seven-Year Cohort Study from a Mexican Tertiary University Hospital

**DOI:** 10.3390/medsci14020214

**Published:** 2026-04-26

**Authors:** Itzel Elizabeth Garibay-Padilla, Jorge Eduardo Hernandez-Del Río, Dayana Estefania Orozco-Sepulveda, Christian Gonzalez-Padilla, Tomas Miranda-Aquino, Vanessa Salas-Bonales, Judith Carolina De Arcos-Jiménez, Jaime Briseño-Ramírez

**Affiliations:** 1 Departamento de Cardiología, Antiguo Hospital Civil de Guadalajara “Fray Antonio Alcalde”, Guadalajara 44280, Mexico; itzelgaribaypadilla@gmail.com (I.E.G.-P.); jehernandezdr@hcg.gob.mx (J.E.H.-D.R.); 2024084@correo.opdhcg.net (D.E.O.-S.); cgonzalez@hcg.gob.mx (C.G.-P.); tmirandaa@hcg.gob.mx (T.M.-A.); vanessasalasbonales@gmail.com (V.S.-B.); 2 Centro Universitario de Tlajomulco, Universidad de Guadalajara, Tlajomulco de Zuñiga 45641, Mexico; judith.dearcos@academicos.udg.mx; 3 Internal Medicine Department, Hospital Civil de Oriente, Tónala 45425, Mexico

**Keywords:** infective endocarditis, in-hospital mortality, prognostic score validation, weighted-incidence syndromic combination antibiogram, Bayesian hierarchical model, antimicrobial stewardship, hemodialysis, Latin America

## Abstract

**Background/Objectives:** Infective endocarditis (IE) carries substantial mortality, particularly in middle-income settings where patient profiles and microbial ecology differ from those of cohorts used to derive international prognostic scores. Syndrome-specific, locally grounded decision aids for empirical therapy are also scarce. We aimed to identify predictors of in-hospital mortality, externally evaluate the RiskE and ICE scores, and construct a Bayesian weighted-incidence syndromic combination antibiogram (WISCA) for IE. **Methods:** We conducted a retrospective cohort study of consecutive adults with definite or possible IE admitted between January 2019 and January 2026. Candidate predictors were screened in two phases, and a clinically specified model was estimated with maximum-likelihood and Firth penalization, with 1000-replicate bootstrap optimism correction. Calibration was assessed with bootstrap calibration plots and the Hosmer–Lemeshow test. Discrimination was compared against RiskE and ICE using DeLong’s test and reclassification metrics. For empirical coverage, we built a WISCA using identified pathogens, reporting both non-Bayesian bootstrap estimates and Bayesian hierarchical partial-pooling estimates with species- and antibiotic-level random intercepts; analyses were also stratified by IE type. **Results:** In-hospital mortality was 22.9% in a young cohort (median 37 years) characterized by high hemodialysis prevalence (47.4%), substantial right-sided IE (46.4%), and *Staphylococcus aureus* predominance (32%) with no methicillin-resistant isolates. Vasopressor-requiring shock (Firth OR 9.23, 95% CI 2.40–40.61) and acute heart failure (OR 10.01, 95% CI 2.78–41.07) were the strongest predictors; the final model achieved an AUC of 0.922 (optimism-corrected 0.908), significantly outperforming RiskE (0.598) and ICE (0.632). The Bayesian WISCA identified multiple carbapenem-sparing and anti-MRSA–sparing regimens with adequate coverage (≥80%), particularly for community-acquired IE, supporting stewardship-oriented empirical selection. Coverage was consistently lower in healthcare-associated IE. **Conclusions:** A parsimonious three-variable model provided strong, locally valid mortality prediction in this hemodialysis-predominant, MRSA-free cohort, substantially outperforming European-derived scores. External validation in independent cohorts is required before clinical adoption. The Bayesian WISCA demonstrated that adequate empirical coverage is achievable without routine broad-spectrum agents, offering institution-specific guidance for stewardship-compatible regimen selection; multicenter validation is warranted.

## 1. Introduction

Infective endocarditis, encompassing all presentations and classifications, remains a significant contributor to global cardiovascular morbidity and mortality [1]. According to the most recent and comprehensive estimates, in 2023 there were approximately 2.34 million disability-adjusted life years (DALYs) and 86,200 deaths attributable to endocarditis worldwide. Infective endocarditis (IE) remains a life-threatening cardiovascular infection with persistently high mortality despite advances in diagnosis, antimicrobial therapy, and cardiac surgery [1]. A recent meta-analysis encompassing 133 studies and over 132,000 patients reported a global short-term mortality of 17%, with Latin America exhibiting the highest regional rate at 33% [2]. Beyond persistently high case-fatality, the absolute global burden of endocarditis continues to rise [3]. Using GBD 1990–2021 estimates, incident cases, deaths, and DALYs increased substantially despite declining age-standardized rates, suggesting that population growth, aging and expanding invasive interventions are key drivers, with a disproportionate burden in older adults and low- and middle-SDI settings [3].

The epidemiological landscape of IE has shifted substantially over the past two decades: in high-income countries (HICs), the typical patient is now elderly with degenerative valve disease or cardiac implantable devices, whereas in low- and middle-income countries (LMICs), younger patients with rheumatic heart disease (RHD) and healthcare-associated risk factors—particularly hemodialysis vascular access—represent an increasingly recognized phenotype [4,5]. Mexican single-center cohorts similarly suggest a high prevalence of healthcare-associated IE and substantial early mortality, reinforcing the need for locally derived prognostic and antimicrobial decision-support tools [6].

Numerous prognostic scores have been developed to predict mortality in IE, including the RiskE [7], ICE-PCS [8], among others. A systematic review identified over 16 published scores incorporating 4 to 18 variables across heterogeneous clinical domains [9]. However, nearly all were derived in European or North American cohorts with demographic profiles that differ markedly from those of LMICs. A recent meta-analysis of 12 scores across 15 validation studies found pooled AUCs ranging from 0.64 to 0.83 with considerable heterogeneity, and concluded that no single score has achieved consistent external validation across diverse populations [10]. However, the geographic transportability of prognostic scores in infective endocarditis is often limited by significant variations in local microbiology and clinical management thresholds, which can compromise their predictive performance in external cohorts, especially in Latin America [11].

Beyond mortality prediction, the selection of appropriate empirical antimicrobial therapy remains a critical challenge in IE management [12]. The high frequency of blood culture-negative IE reported in Latin America increases uncertainty in early management and heightens reliance on empiric regimens that must balance adequate coverage with antimicrobial stewardship [12]. ESC guidance emphasizes obtaining three sets of blood cultures before antibiotics and starting empiric intravenous therapy promptly, with rapid adaptation to targeted therapy once the causative pathogen and susceptibility are identified [13]. In real-world cohorts, unsuitable antibiotic therapy has been independently associated with higher in-hospital mortality, supporting the clinical value of tools that improve empiric regimen selection in IE [14].

The weighted-incidence syndromic combination antibiogram (WISCA) is a methodology that integrates local pathogen prevalence with organism-specific susceptibility to estimate the probability that a given antimicrobial regimen will cover the causative pathogen of a clinical syndrome [15]. While WISCA has been applied to several infectious syndromes, evidence in infective endocarditis remains scarce and largely limited to recent single-center experiences; to our knowledge, Bayesian WISCA frameworks and data from Latin America are still lacking [16,17,18]. Because syndrome- and strata-specific susceptibility estimates can be sparse, Bayesian implementations of WISCA allow principled borrowing of strength across strata and provide uncertainty quantification for regimen-level coverage estimates [19]. Given the diverse microbial ecology of IE—spanning staphylococci, streptococci, enterococci, gram-negative organisms, and fungi—a syndrome-specific weighted antibiogram could provide valuable guidance for empirical therapy selection, particularly in settings when a well-defined syndrome arises from heterogeneous etiologic agents across heterogeneous patient populations, within a locally specific resistance ecology that makes “one-size-fits-all” empiric regimens unreliable [19,20,21].

Taken together, the high regional mortality, frequent diagnostic uncertainty (including culture-negative IE), and locally variable pathogen distribution and resistance patterns underscore the need for (i) context-specific risk stratification and external validation of widely used prognostic scores, and (ii) quantitative decision support for empiric therapy that is explicitly grounded in local microbiology. Therefore, we aimed to identify independent predictors of in-hospital mortality and develop an internally validated model, externally validate RISK-E and ICE scores in a Mexican cohort, and construct a Bayesian WISCA to inform empiric antimicrobial selection in IE.

## 2. Materials and Methods

### 2.1. Study Design, Setting, and Population

Hospital Civil de Guadalajara “Fray Antonio Alcalde” is a 1000-bed public tertiary university hospital and the primary referral center for western Mexico. Patients with infective endocarditis (IE) at this institution exhibit a distinctive epidemiological profile characterized by younger age, a high prevalence of hemodialysis, and a predominance of healthcare-associated IE features that differ substantially from the populations in which international prognostic scores were originally derived. Accordingly, we conducted a single-center retrospective cohort study including all consecutive adult patients (≥18 years) with definite or possible IE according to the modified Duke criteria [22], diagnosed from January 2019 to January 2026. Possible IE cases were included because the modified Duke criteria explicitly recognize this category as warranting treatment, and exclusion would introduce selection bias against patients with incomplete diagnostic workup who nonetheless received a clinical diagnosis of IE. The study was approved by the institutional ethics committee (CEI 343/25, 28 January 2026) and classified as minimal-risk research, with a waiver of informed consent due to its retrospective design.

### 2.2. Data Collection and Variables

Clinical, microbiological, echocardiographic, and outcome data were extracted from electronic medical records into a standardized database comprising 161 variables. Variables were structured a priori into domains encompassing demographics (age, sex, BMI, Charlson comorbidity index), comorbidities (diabetes, chronic kidney disease, hepatopathy, active neoplasia, HIV with immunosuppression, intravenous drug use), healthcare-associated risk factors (prior endocarditis, prosthetic valve, central venous/Mahurkar catheter, cardiac implantable electronic device, hemodialysis), infective endocarditis (IE) classification (acquisition category, grouped acquisition, prosthetic vs. native valve, and affected side), microbiology (blood-culture positivity, pathogen group, and organism identification), echocardiography (LVEF and categories, vegetation characteristics including size, number, mobility, and thresholds >10 mm and >30 mm), complications (clinical and structural complications and total complication count), treatment (surgery and surgical indications, empirical regimen, and appropriateness of initial therapy), prognostic scores (RiskE [7], ICE [8], and SOFA-2 Score [23] computed per published algorithms), and antimicrobial susceptibility.

Seven derived variables were created for the extended analysis: *S. aureus* as causative pathogen, culture-negative IE, left-sided IE, structural complication (abscess, perforation, fistula, or aneurysm), reduced LVEF (<50%), high comorbidity burden (Charlson ≥ 4), and absence of initial clinical suspicion of IE (defined as IE not being among the admission diagnoses documented at hospital entry).

### 2.3. Outcomes

The primary outcome was in-hospital all-cause mortality, defined as death from any cause during the index hospitalization; this endpoint was available for 96 of 97 patients (one missing value). Secondary outcomes comprised 30-day all-cause mortality (available for 86 of 97 patients) and embolic events. Exploratory outcomes included ICU admission, prolonged hospital stay (defined as exceeding the 90th percentile, >69 days), and a composite endpoint of complicated IE (vasopressor-requiring shock, acute heart failure, embolism, or ICU admission). Multivariable models for these exploratory outcomes were developed using the same analytical framework as the primary model (bivariate screening, backward AIC, and Firth penalization) and are reported in the Supplementary Material (Supplementary Tables S2–S5).

### 2.4. Predictive Model Development

#### 2.4.1. Variable Selection

Variable selection followed a hybrid strategy combining clinical domain knowledge with statistical screening, as recommended for small-sample prediction modeling [24,25]. Rather than relying solely on univariate screening—which we acknowledge has limitations including potential overfitting and biased coefficient estimation [26]—we used a two-phase approach in which clinical plausibility and domain representation guided both candidate identification and final model specification. The variable originally recorded as “septic shock” in clinical charts was reclassified as “vasopressor-requiring shock,” as hemodynamic collapse in IE frequently involves mixed cardiogenic and distributive mechanisms that cannot be reliably distinguished retrospectively; the use of vasoactive agents (norepinephrine, vasopressin, or dobutamine) to maintain hemodynamic stability represents an objective, reproducible criterion independent of the presumed shock etiology.

In Phase 1, 27 candidate variables across nine clinical domains were screened using univariate logistic regression; variables with p<0.20 (Hosmer–Lemeshow criterion) were retained as candidates. In Phase 2, an extended bivariate analysis evaluated 35 additional variables, including the eight derived variables, using the same threshold. Quasi-complete separation was handled by reporting Fisher’s exact test *p*-values, and multilevel categorical variables were evaluated using likelihood ratio tests. The screening results were used to identify candidate predictors from each clinical domain, not to mechanically select the final model.

#### 2.4.2. Model Specification

The final model was specified based on three criteria: (1) representation of three independent clinical domains (hemodynamic: vasopressor-requiring shock; anatomic: left-sided IE; cardiac complication: acute heart failure); (2) statistical significance in univariate analysis (all three p<0.05); and (3) adherence to the events-per-variable (EPV) constraint (22 events/3 predictors = EPV 7.3). Multicollinearity was assessed using variance inflation factors (VIF < 2 for all predictors).

The variable “high-risk pathogen” was evaluated and excluded after stratified analysis revealed massive confounding: coagulase-negative staphylococcal mortality was entirely attributable to left-sided IE (4/4 deaths were left-sided), and the paradoxically low *S. aureus* mortality (12.9%) was explained by the predominance of right-sided IE among *S. aureus* cases (58%). Age was formally evaluated as a fourth predictor and excluded based on a non-significant likelihood ratio test (p=0.054), marginal AIC improvement (−1.7), increase in bootstrap optimism (0.014→0.017), destabilization of left-sided IE (p=0.182→0.062), and decrease in EPV from 7.3 to 5.5.

#### 2.4.3. Model Estimation and Penalization

The primary model was estimated using standard maximum likelihood logistic regression. Given the moderate EPV (7.3), Firth’s penalized logistic regression was performed as the primary sensitivity analysis to reduce small-sample bias [27]. Consistency between standard and Firth-penalized estimates was assessed by comparing odds ratios, confidence intervals, and *p*-values.

#### 2.4.4. Confirmatory LASSO Analysis

To assess the robustness of variable selection independently of the screening-based approach, a LASSO logistic regression (L1-penalized) was performed using 10-fold cross-validated glmnet on 18 candidate variables. Results at both $\lambda_{\min}$ and $\lambda_{1\mathrm{SE}}$ were compared with the final model. LASSO at $\lambda_{1\mathrm{SE}}$ retained vasopressor-requiring shock and acute heart failure; at $\lambda_{\min}$ it retained all three predictors plus additional variables, providing independent confirmation of the core model composition. Individual crude odds ratios for all screened variables are provided in Supplementary Table S7; a comprehensive multi-model comparison is presented in Supplementary Table S8.

#### 2.4.5. Internal Validation

Internal validation was performed using bootstrap optimism correction (1000 replicates) as described by Harrell [25]. In each replicate, the model was fit on a bootstrap sample and evaluated on both the bootstrap sample (apparent performance) and the original sample; optimism was estimated as the average difference. The corrected AUC was computed as ${\mathrm{AUC}}_{\mathrm{apparent}}-\mathrm{optimism}$. Model calibration was assessed using the Hosmer–Lemeshow goodness-of-fit test, bootstrap calibration plots with calibration intercept and slope, and the Brier score.

#### 2.4.6. Missing Data

Missing data were characterized using variable-level completeness summaries and Little’s MCAR test. The three model predictors (vasopressor-requiring shock, left-sided IE, acute heart failure) each had ≤1.0% missing data (1/97), and the primary outcome had 1.0% missing. Complete-case analysis (CCA) was used as the primary approach. Multiple imputation by chained equations (MICE; m=20, CART method, 10 iterations) was performed as a prespecified sensitivity analysis; pooled estimates were obtained using Rubin’s rules. The MICE analysis yielded virtually identical odds ratios and AUC, confirming that CCA was appropriate given the minimal and likely MCAR missingness pattern.

#### 2.4.7. Exploratory Embolism Model

An exploratory predictive model for embolic events was developed using the same analytical framework (bivariate screening with p<0.20, backward AIC selection, Firth penalization, and 1000-replicate bootstrap). The variable “vascular/immunological phenomena” was excluded a priori due to definitional overlap with the outcome. Vegetation characteristics (size > 10 mm, mobility, size > 30 mm) were included as candidate predictors.

### 2.5. External Validation of Prognostic Scores

The RiskE [7] and ICE [8] scores were calculated according to published algorithms. The SOFA-2 score was computed using the updated Sequential Organ Failure Assessment (SOFA-2) criteria [23], which incorporate revised cardiovascular, renal, and coagulation components compared with the original SOFA score. Discrimination was assessed using the area under the receiver operating characteristic curve (AUC-ROC). Head-to-head comparison of the local model against each score was performed using DeLong’s test for correlated ROC curves [28]. Reclassification improvement was quantified using net reclassification improvement (NRI) and integrated discrimination improvement (IDI) [29].

### 2.6. Bayesian Weighted-Incidence Antibiogram

#### 2.6.1. Study Population and Pathogen Distribution

The WISCA analysis included all patients with an identified pathogen (n=68), excluding only culture-negative IE (n=29). Twenty-three distinct bacterial species were identified and used as individual grouping levels for the hierarchical model. The four fungal isolates were retained in the dataset with all antibacterial susceptibilities set to zero, ensuring their prevalence was correctly captured in the weighted coverage calculation. Antimicrobial susceptibility testing was performed routinely by the hospital microbiology laboratory using the VITEK 2 system (bioMérieux, Marcy-l’Étoile, France) according to current CLSI breakpoints, and results were extracted for 20 antibacterial agents. A total of 54 regimens were evaluated: 20 monotherapies and 34 two-drug combinations, with combination susceptibility defined by OR logic (susceptible if at least one agent was active). Combinations were selected based on current IE treatment guidelines [13] and institutional prescribing patterns, encompassing beta-lactam + aminoglycoside, beta-lactam + glycopeptide/lipopeptide/oxazolidinone, and glycopeptide + aminoglycoside regimens commonly used in clinical practice.

#### 2.6.2. Bayesian Hierarchical Model

The WISCA methodology estimates the probability that a given empirical regimen covers the causative pathogen of a clinical syndrome, weighted by local pathogen prevalence [30]. For each regimen *r*, the global weighted coverage $C_{r}$ is computed as:

(1) $$C_{r}=\sum_{s=1}^{S}\pi_{s}\;\cdot \;P\left(\mathrm{susceptible}\;\mathrm{to}\;r|\mathrm{species}=s\right)$$

where $\pi_{s}$ is the prevalence of species *s* among IE pathogens, S=23 species, and P(susceptible) is derived from the posterior distribution of the Bayesian model.

A Bayesian hierarchical logistic regression model was fit using the brms package [31] with the cmdstanr backend:

(2) $${\mathrm{susceptible}}_{ij}∼\mathrm{Bernoulli}\left({\mathrm{logit}}^{-1}\left(\eta_{ij}\right)\right)$$

(3) $$\eta_{ij}=\beta_{0}+\beta_{1}\;\cdot \;{\mathrm{age}}_{i}+\beta_{2}\;\cdot \;{\mathrm{sex}}_{i}+u_{\mathrm{species}[i]}+v_{\mathrm{antibiotic}[j]}$$

where $u_{\mathrm{species}}∼N\left(0,\sigma_{\mathrm{species}}^{2}\right)$ and $v_{\mathrm{antibiotic}}∼N\left(0,\sigma_{\mathrm{antibiotic}}^{2}\right)$ are random intercepts providing partial pooling across rare species and antibiotics.

Weakly informative Student-t(3,0,2.5) priors were placed on the intercept and random-effect standard deviations; N(0,1) priors were placed on fixed effects. Four MCMC chains were run for 4000 iterations each (2000 warmup), yielding 8000 posterior samples. Convergence was assessed using $\hat{R}$ (all ≤1.005) and divergent transition counts (zero).

For combination regimens, species-level coverage was derived using the OR independence assumption:

(4) P(coveredbyA+B∣s)=1−[1−P(A∣s)]×[1−P(B∣s)]

Ninety-five percent highest density intervals (HDI) were computed from the posterior distribution. A non-hierarchical (traditional) WISCA was computed in parallel using bootstrap confidence intervals (B=2000) for comparison.

#### 2.6.3. Stratified Analysis

A second model incorporated IE type (community-acquired vs. healthcare-associated) as a fixed effect to estimate stratum-specific coverage. Correlation between strata was quantified using Pearson’s correlation coefficient of posterior median coverages.

### 2.7. Statistical Analysis

Continuous variables were summarized as median (interquartile range [IQR]) or mean ± standard deviation as appropriate based on the Shapiro–Wilk test for normality. Categorical variables were expressed as absolute frequencies and percentages. Between-group comparisons used the Wilcoxon rank-sum test for continuous variables and the chi-squared test or Fisher’s exact test for categorical variables. A two-sided significance level of α=0.05 was used throughout. No correction for multiple comparisons was applied in the bivariate screening phase, consistent with the Hosmer–Lemeshow strategy of using a liberal threshold p<0.20 for candidate identification, with subsequent validation through penalized methods and bootstrap internal validation.

Complete-case analysis (CCA) was used as the primary analytical approach, with MICE-based multiple imputation as a sensitivity analysis. Missing data mechanisms were evaluated using the Missing Completely at Random (MCAR) test of Little, missing data patterns were visualized using aggregation and combination plots, and the proportion of missing values was computed for each variable.

### 2.8. Software

All analyses were performed in R version 4.4.1 (R Foundation for Statistical Computing, Vienna, Austria). Key R packages included: tidyverse (v2.0.0) for data manipulation and visualization; readxl (v1.4.5) for Excel file import; gtsummary (v2.5.0) for publication-ready summary tables; pROC (v1.19.0.1) for ROC curve analysis and DeLong’s test; rms (v8.1.1) for regression modeling, bootstrap validation, calibration, and nomograms; logistf (v1.26.1) for Firth’s penalized logistic regression; glmnet (v4.1.10) for LASSO regression; mice (v3.19.0) for multiple imputation by chained equations; flextable (v0.9.11) and officer (v0.7.3) for Word document export; brms (v2.22.0) for Bayesian hierarchical models via Stan; cmdstanr (v0.9.0) as the Stan backend for MCMC sampling; HDInterval (v0.2.4) for highest density interval computation; bayesplot (v1.15.0) and posterior (v1.7.0) for MCMC diagnostics; and dcurves (v0.5.1) for decision curve analysis. The complete analysis pipeline is available from the corresponding author upon request.

## 3. Results

### 3.1. Study Population

Ninety-seven patients met inclusion criteria: 89 (91.8%) with definite IE (60 pathological, 29 clinical) and 8 (8.2%) with possible IE according to the modified Duke criteria. The median age was 37 years (IQR 28–52; range 16–80), and 73 (75.3%) were male. The median Charlson comorbidity index was 2 (IQR 0–3). The most prevalent comorbidity was chronic kidney disease requiring hemodialysis (46/97, 47.4%), followed by central venous catheter (44/97, 45.4%), chronic kidney disease (43/97, 44.3%), recent hospitalization (43/97, 44.3%), and diabetes mellitus (24/97, 24.7%). Prosthetic valve IE accounted for 11.3% (11/97), and only 3.1% (3/97) reported intravenous drug use.

Healthcare-associated IE predominated (52/97, 53.6%) over community-acquired IE (42/97, 43.3%). Native valve IE accounted for 88.7% (86/97). Left-sided IE was present in 49.5% (48/97) and right-sided in 46.4% (45/97), with 2.1% bilateral. Clinical characteristics differed substantially between IE types: community-acquired IE was characterized by higher prevalence of *Streptococcus* spp. and left-sided involvement, while healthcare-associated IE was dominated by *S. aureus*, hemodialysis access (86.5% vs. 0%), and central venous catheter use; in-hospital mortality did not differ significantly between groups (28.6% vs. 17.3%; p=0.220) (Supplementary Table S1). In-hospital survival data were available for 96 patients; Table 1 summarizes clinical characteristics stratified by survival status.

### 3.2. Microbiology

**Figure 1:**
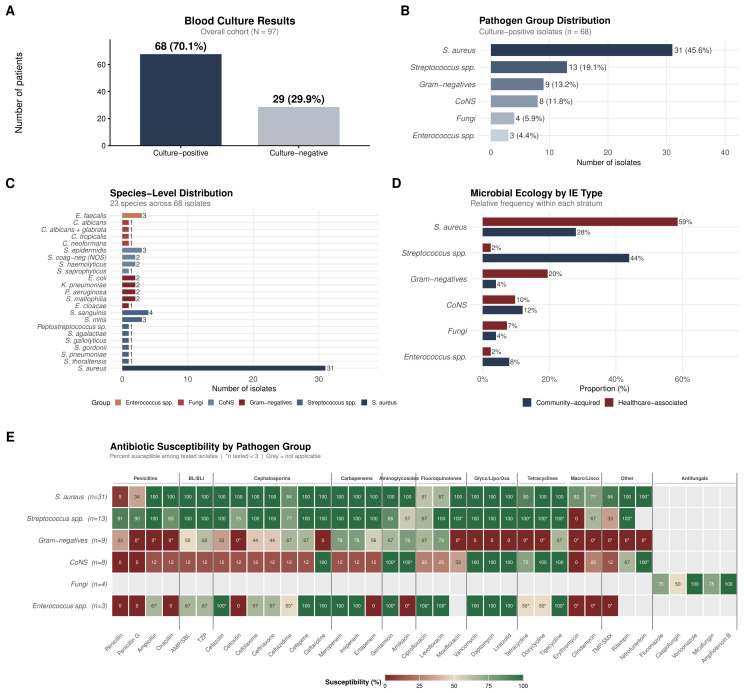
Comprehensive microbiological profile of infective endocarditis (N=97). (**A**) Blood culture results: 68 culture-positive (70.1%) and 29 culture-negative (29.9%). (**B**) Pathogen group distribution among culture-positive isolates (n=68). (**C**) Species-level breakdown: 23 distinct species identified across six pathogen groups. (**D**) Microbial ecology stratified by IE type (community-acquired vs. healthcare-associated), illustrating the dominance of *Streptococcus* spp. in community-acquired and *S. aureus* in healthcare-associated IE. (**E**) Antibiotic susceptibility heatmap (percent susceptible) by pathogen group across all tested antimicrobial agents, organized by pharmacological class. In Panel (**E**), asterisks (*) indicate antibiotic–pathogen combinations with fewer than 3 isolates tested (n<3); grey cells denote combinations not applicable to the pathogen group (e.g., antifungal agents tested only against fungal isolates).

Blood cultures were positive in 70.1% (68/97) of patients (Figure 1A). *Staphylococcus aureus* was the most common pathogen (31/97, 32.0%), followed by culture-negative IE (29/97, 29.9%), *Streptococcus* spp. (13/97, 13.4%), gram-negative organisms (9/97, 9.3%), coagulase-negative staphylococci (8/97, 8.2%), fungi (4/97, 4.1%), and *Enterococcus* spp. (3/97, 3.1%; Figure 1B,C). In-hospital mortality varied markedly by pathogen group: *Enterococcus* spp. exhibited 100% mortality (3/3), fungi 50% (2/4), coagulase-negative staphylococci 50% (4/8), culture-negative IE 20.7% (6/29), *Streptococcus* spp. 16.7% (2/12), *S. aureus* 12.9% (4/31), and gram-negative organisms 11.1% (1/9). The overall association between pathogen group and mortality was statistically significant (likelihood ratio test p=0.013). These pathogen-specific mortality estimates should be interpreted with caution given the very small subgroup sizes (e.g., *Enterococcus* n=3, fungi n=4), which preclude reliable inference. Notably, *S. aureus* mortality was paradoxically low (12.9%) compared with international reports of 20–40%, a finding attributable to the predominance of right-sided IE among *S. aureus* cases (58%), which carries an inherently better prognosis.

The microbial ecology differed between IE types: *Streptococcus* spp. dominated community-acquired IE (11/24, 45.8%), while *S. aureus* dominated healthcare-associated IE (24/38, 63.2%), with a notable proportion of gram-negative organisms in the latter (8/38, 21.1%) (Figure 1D). Antibiotic susceptibility patterns by pathogen group are summarized in Figure 1E.

### 3.3. Echocardiography and Complications

Transthoracic or transesophageal echocardiography data were available for 94 of 97 patients (96.9%). Median left ventricular ejection fraction (LVEF) was 60% (IQR 55–63), with preserved function (LVEF ≥ 50%) in 85.1% (80/94), moderately reduced function (40–49%) in 10.6% (10/94), and reduced function (<40%) in 4.3% (4/94). Vegetations were detected in 83.5% (81/97) of patients, with a median maximal dimension of 19 mm (IQR 13–24); vegetations exceeding 10 mm were observed in 85.2% (69/81) of those with measurable lesions. The median number of vegetations was 1 (range 1–3): 62 patients had a single vegetation, 17 had two, and 2 had three.

**Figure 2:**
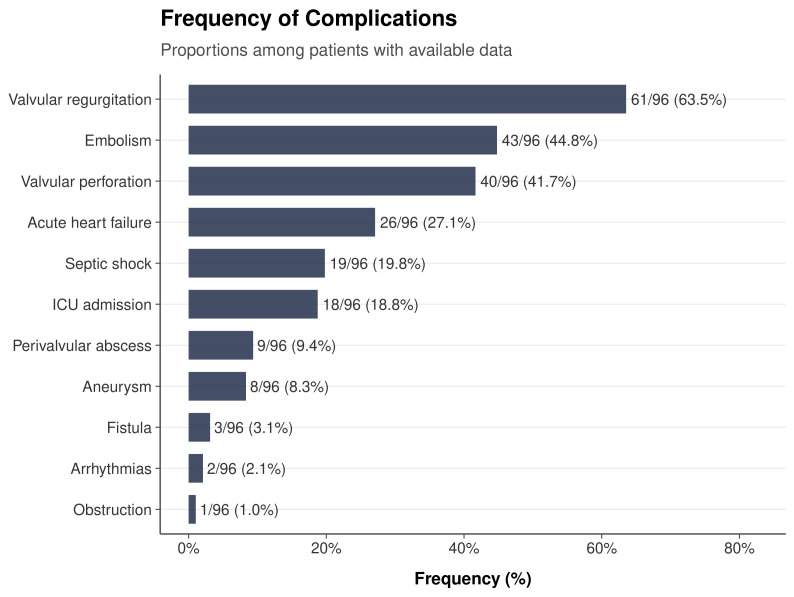
Complication profile of infective endocarditis cases (N=96). Horizontal bar chart showing the frequency and percentage of each complication.

The most frequent complications (Figure 2) were valvular regurgitation (61/96, 63.5%), embolism (43/96, 44.8%), valvular perforation (40/96, 41.7%), acute heart failure (26/96, 27.1%), vasopressor-requiring shock (19/96, 19.8%), and ICU admission (18/96, 18.8%). Less common complications included perivalvular abscess (9/96, 9.4%), aneurysm (8/96, 8.3%), fistula (3/96, 3.1%), arrhythmias (2/96, 2.1%), and obstruction (1/96, 1.0%). The median total number of complications was 2 (IQR 1–3).

### 3.4. Outcomes and Treatment

In-hospital mortality was 22.9% (22/96; 95% CI 15.0–32.6%), and 30-day all-cause mortality was 27.9% (24/86; 95% CI 18.8–38.6%). Non-survivors were significantly older than survivors (median 50.5 vs. 34 years; p=0.002). Surgical intervention was performed in 63.2% (60/95; 95% CI 52.6–72.8%) of patients; among those with a documented surgical indication, 100% (59/59) underwent surgery, indicating complete adherence to surgical guidelines. ICU admission occurred in 18.8% (18/96), and vasopressor-requiring shock in 19.8% (19/96). Acute heart failure developed in 21.9% (21/96), and embolic events were documented in 44.8% (43/96). Median hospital stay was 34.5 days (IQR 25.8–50.3), with no significant difference between survivors (34 days, IQR 26–50) and non-survivors (36 days, IQR 21.5–51.5).

Prognostic scores differed between survivors and non-survivors: median RiskE was 9 points (IQR 0–9) in survivors vs. 9 (IQR 7–14) in non-survivors; median ICE score was 7 (IQR 5–9) vs. 9 (IQR 6–11); and median SOFA-2 score was 4 (IQR 0–4) vs. 3 (IQR 1–4). The ICE score was significantly higher in non-survivors (Wilcoxon p=0.017), while RiskE showed a trend (p=0.064) and the SOFA-2 score was not significantly different (p=0.146).

Empirical antimicrobial therapy was highly heterogeneous but predominantly centered on broad-spectrum β-lactam plus anti-MRSA coverage. The most frequently prescribed regimen was ceftriaxone + vancomycin (35/95, 36.8%), followed by ceftazidime + vancomycin (8/95, 8.4%) and ceftriaxone + linezolid (5/95, 5.3%); all other regimens were each used in ≤4.2% of cases, reflecting substantial variability in initial management. Combination regimens clearly predominated over monotherapy. Overall empirical therapy appropriateness was 83.6% (46/55 evaluable cases), with a median time to antimicrobial adjustment of 3.5 days (IQR 3–6.75). The complete list of empirical antimicrobial regimens is shown in Supplementary Table S6.

In multivariable logistic regression models for secondary outcomes (Supplementary Table S5), ICU admission was independently associated with both vasopressor-requiring shock and acute heart failure, with consistent effect sizes in standard and Firth-penalized models (Firth OR 7.03, 95% CI 1.99–25.90; p=0.003 and Firth OR 7.05, 95% CI 2.09–25.61; p=0.002, respectively), with an exploratory model AUC of 0.874 (Supplementary Table S2). Prolonged hospitalization (>69 days; P90), occurred in 9.4% (9/96) of patients and, in bivariate comparisons, was associated with female sex (55.6% vs. 21.8%; p=0.041) and greater acute illness severity at presentation, reflected by higher SOFA-2 scores (median 4.0 [IQR 4.0–5.0] vs. 2.5 [0.0–4.0]; p=0.017), whereas other baseline characteristics, IE classification variables, echocardiographic features, and management factors did not show statistically significant differences (Table S3).

In contrast, a composite endpoint of complicated IE (vasopressor-requiring shock, acute heart failure, embolism, or ICU admission) was frequent (59/96) and was associated with older age (median 46.0 vs. 33.0 years; p=0.039), community-acquired presentation (54.4% vs. 29.7%; p=0.019), and—most strongly—left-sided involvement (67.8% vs. 21.6%; p<0.001), alongside higher RiskE scores (median 9.0 [5.0–14.0] vs. 9.0 [0.0–9.0]; p=0.039) and a markedly higher frequency of fulfilled surgical indication (88.5% vs. 48.1%; p<0.001); mortality clustered in the complicated group (37.3% vs. 0%; p<0.001) (Table S4).

### 3.5. Predictive Model for In-Hospital Mortality

#### 3.5.1. Bivariate Analysis

A comprehensive two-phase bivariate screening was performed to identify candidate predictors of in-hospital mortality. In Phase 1, 27 variables across nine original clinical domains (demographics, comorbidities, risk factors, IE classification, microbiology, echocardiography, complications, treatment, and prognostic scores) were evaluated using univariate logistic regression. In Phase 2, an additional 35 variables were screened, including eight derived variables (left-sided IE, *S. aureus* pathogen, high-risk pathogen, culture-negative IE, structural complication, reduced LVEF, high comorbidity burden, and absence of initial clinical suspicion) and variables not evaluated in Phase 1 (individual complications, clinical presentation features, biomarkers, temporal variables, and additional risk factors). Table 2 presents the bivariate screening organized by 16 clinical domains; an extended version of crude odds ratios for in-hospital mortality is depicted in Supplementary Table S7.

Of the 62 variables screened, 12 reached conventional statistical significance (p<0.05) and 25 met the Hosmer–Lemeshow criterion for candidate retention (p<0.20). The strongest bivariate associations with in-hospital mortality were observed for:

Hemodynamic and clinical complications: Vasopressor-requiring shock exhibited the strongest crude association (OR 16.37, 95% CI 5.21–57.90, p<0.001), followed by acute heart failure (OR 8.67, 95% CI 2.98–26.86, p<0.001), and ICU admission (OR 5.00, 95% CI 1.67–15.35, p=0.004). The composite variable of total complications per unit increment was also significant (OR 2.20, 95% CI 1.53–3.40, p<0.001).

IE classification: Affected side was significantly associated with mortality (LRT p=0.005), with left-sided IE conferring substantially higher risk. IE type (community-acquired vs. healthcare-associated vs. nosocomial) also showed a significant global association (LRT p=0.011). The derived variable left-sided IE as a binary predictor was significant (OR 3.24, 95% CI 1.14–9.86, p=0.030).

Demographics: Age per year (OR 1.04, 95% CI 1.01–1.08, p=0.005) and Charlson comorbidity index per point (OR 1.30, 95% CI 1.02–1.66, p=0.032) were significant continuous predictors.

Prognostic scores: The ICE score was significant (OR 1.20 per point, 95% CI 1.04–1.42, p=0.017), while RiskE showed a trend (OR 1.05, 95% CI 1.00–1.12, p=0.064).

Quasi-complete separation was observed for arrhythmias (2/2 patients with arrhythmias died; Fisher p=0.051) and surgical indication (Fisher p=0.002); crude odds ratios were not estimable for these variables. No comorbidities (diabetes, chronic kidney disease, hepatopathy, neoplasia, intravenous drug use), healthcare exposure variables (hemodialysis, central venous catheter), biomarkers (lactate, procalcitonin), or temporal variables (symptom duration, time to antimicrobial correction) were significantly associated with in-hospital mortality.

#### 3.5.2. Multivariate Analysis

Two local predictive models were developed and compared head-to-head alongside international prognostic scores, following TRIPOD guidelines for transparent reporting [26].

Model 1 (Backward AIC). Backward stepwise selection by AIC from Phase 1 bivariate candidates retained four predictors—age, vasopressor-requiring shock, acute heart failure, and surgery (4 predictors; EPV = 5.5). This model achieved an apparent AUC of 0.933 (95% CI, 0.88–0.99), with a bootstrap optimism-corrected AUC of 0.916.

Model 2 (Final Clinical Model). A clinically guided three-variable model incorporating vasopressor-requiring shock, left-sided IE, and acute heart failure (3 predictors; EPV = 7.3) was selected as the final specification based on superior EPV, parsimony, and multi-domain clinical representation.

DeLong’s test showed no significant difference between Model 1 and Model 2 (z=−0.86, p=0.388), supporting the selection of the more parsimonious Model 2 with equivalent discrimination and lower risk of overfitting. The final three-variable model and its Firth-penalized estimates are shown in Table 3 and Table 4.

#### 3.5.3. Final Model

Vasopressor-requiring shock and acute heart failure were the strongest independent predictors of in-hospital mortality (Table 4), with Firth-penalized ORs of 9.23 (95% CI, 2.40–40.61; p=0.001) and 10.01 (95% CI, 2.78–41.07; p<0.001), respectively—each consistent with an approximately 10-fold increase in the odds of death. Left-sided IE showed a non-significant trend toward increased mortality (Firth OR 2.66; 95% CI, 0.62–12.68; p=0.185) and was retained based on established prognostic relevance and contribution to multi-domain clinical representation.

Model 2 demonstrated excellent discrimination, with an apparent AUC of 0.922 (95% CI, 0.87–0.98). Internal validation using 1000 bootstrap replicates yielded an optimism-corrected AUC of 0.908 (optimism = 0.014), indicating minimal overfitting. Additional performance metrics included an AIC of 64.8, Hosmer–Lemeshow p=0.505, Nagelkerke pseudo-$R^{2}=0.583$, and a Brier score of 0.092 (below the trivial Brier of 0.177), supporting adequate calibration and overall fit. Variance inflation factors were <2 for all predictors, excluding clinically meaningful multicollinearity.

Firth-penalized estimation produced the expected attenuation of odds ratios relative to maximum likelihood while preserving the direction and statistical significance of predictors, supporting robustness under an EPV of 7.3 (22 events/3 predictors). In confirmatory regularization, LASSO at $\lambda_{1\mathrm{SE}}$ retained vasopressor-requiring shock and acute heart failure as the only non-zero predictors; at $\lambda_{\min}$, all three final model predictors were retained alongside additional variables, consistent with the core model structure. A comprehensive multi-model comparison including all fitted models, LASSO specifications, and international scores is provided in Supplementary Table S8.

**Figure 3:**
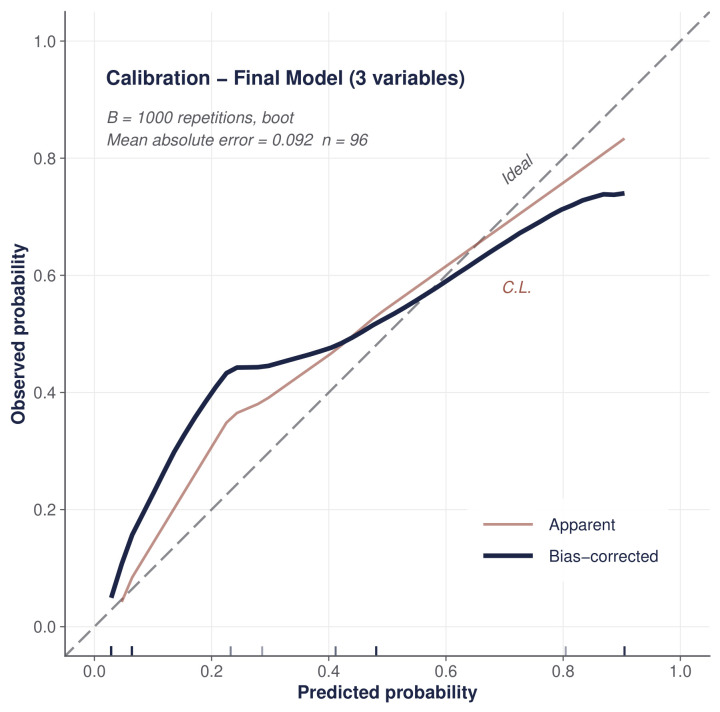
Bootstrap calibration plot for the primary mortality prediction model (B=1000). The solid line represents the bias-corrected calibration curve; the dashed line represents ideal calibration. Hosmer–Lemeshow p=0.505.

The bootstrap calibration plot (Figure 3) confirmed adequate model calibration, with the calibration curve closely following the ideal diagonal line.

Multiple imputation sensitivity analysis (MICE, m=20, CART method) yielded virtually identical odds ratios and model discrimination (Table 5): vasopressor-requiring shock OR 11.12 (95% CI 2.47–50.11), left-sided IE OR 2.87 (95% CI 0.58–14.23), and acute heart failure OR 12.28 (95% CI 2.92–51.60), with a pooled AUC of 0.922—confirming that CCA was appropriate given the minimal and likely MCAR missingness pattern.

Sensitivity analysis adding age as a fourth predictor (Model 2 + age) yielded a marginal increase in apparent AUC (0.937 vs. 0.922), but the likelihood ratio test was marginally non-significant (p=0.054). AIC improved by only 1.7 units (64.8 to 63.1), bootstrap optimism increased (0.014→0.017), and left-sided IE was destabilized (p=0.182→0.062). EPV declined from 7.3 to 5.5, increasing overfitting risk; therefore, the three-variable model was retained as final.

#### 3.5.4. Comparison with International Scores

**Figure 4:**
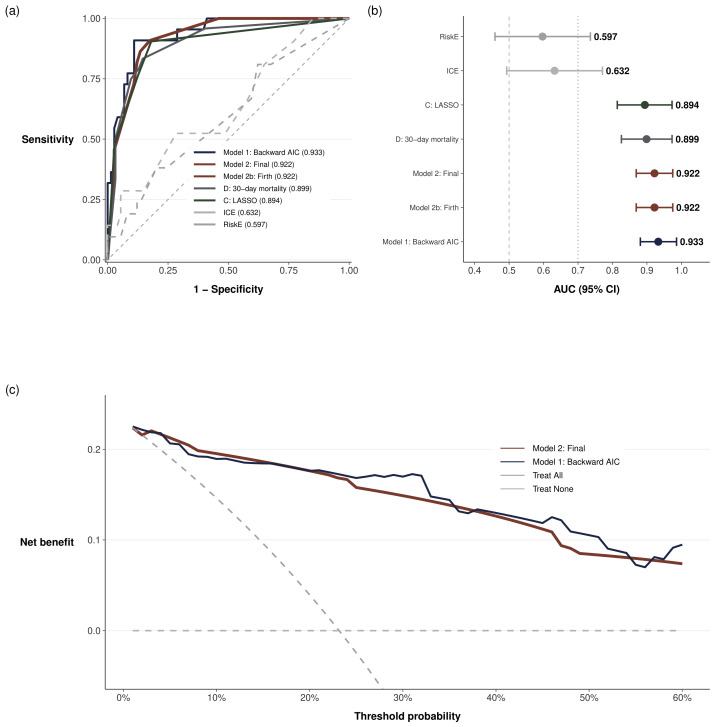
Predictive performance of local and international models for in-hospital mortality in infective endocarditis. (**a**) Receiver operating characteristic curves for all evaluated models; the final three-variable model (Model 2) is highlighted. (**b**) Forest plot of area under the curve with 95% confidence intervals, ordered by discriminative performance; vertical dashed lines indicate non-discrimination (0.5) and acceptable discrimination (0.7) thresholds. (**c**) Decision curve analysis comparing the net clinical benefit of Model 2 and Model 1 against default strategies (treat all, treat none) across threshold probabilities of 0–60%.

The local model significantly outperformed both international prognostic scores (Table 6, Figure 4b). RiskE achieved an AUC of 0.598 (95% CI, approximately 0.45–0.74) and ICE an AUC of 0.632 (95% CI, approximately 0.49–0.77), both approaching non-discrimination and statistically indistinguishable (DeLong z=−0.55, p=0.581). DeLong’s test confirmed that Model 2 significantly outperformed RiskE (z=4.69, p<0.0001) and ICE (z=4.08, p<0.0001), with absolute AUC differences of 0.324 and 0.290, respectively; Model 1 showed similar superiority (p<0.0001 for both comparisons).

Net reclassification improvement supported superior reclassification by the local model (NRI vs. RiskE = 0.761; 95% CI 0.29–1.20; p=0.001; NRI vs. ICE = 0.978; 95% CI 0.52–1.41; p<0.0001). Integrated discrimination improvement was significant for both comparisons (IDI vs. RiskE = 0.433, p<0.001; IDI vs. ICE = 0.404, p<0.001), indicating improved separation of predicted probabilities between events and non-events.

The poor performance of international scores likely reflects a population mismatch. RiskE was derived from left-sided IE patients undergoing cardiac surgery in Spanish centers, whereas 46.4% of the present cohort had right-sided IE. Neither score incorporates hemodialysis status, vascular access type, or healthcare-associated IE—key determinants of risk in this setting. Concentrated score distributions (similar medians in survivors and non-survivors) further suggest limited ability to capture the principal axes of prognostic variability in this cohort.

Decision curve analysis indicated that the local model provided greater net clinical benefit than treat-all or treat-none strategies across threshold probabilities of approximately 5–50%, supporting potential clinical utility for risk-stratified decision-making (Figure 4c).

#### 3.5.5. Secondary Model: 30-Day Mortality

The same three-predictor model applied to 30-day mortality (n=86, 24 events, EPV 8.0) achieved an AUC of 0.899 (95% CI 0.83–0.97; optimism-corrected 0.888; Hosmer–Lemeshow p=0.127), confirming the robustness of the predictors across time horizons.

#### 3.5.6. Exploratory Model for Embolic Events

An exploratory predictive model for embolic events was developed as a secondary analysis. Among 93 evaluable patients, 42 (45.2%) experienced embolic events. Bivariate screening of 42 variables identified 11 candidates (p<0.20), of which 5 reached conventional significance. The variable “vascular/immunological phenomena” was excluded due to definitional overlap with the outcome. Notably, vegetation characteristics—size > 10 mm, mobility, and size > 30 mm—were not significant predictors of embolism (all p>0.25).

The final backward AIC model (Model E) and its Firth-penalized estimates are shown in Table 7. A clinical a priori model (IE type + left-sided IE + vegetation > 10 mm) achieved a lower AUC of 0.663 (optimism-corrected 0.626); DeLong comparison was non-significant (z=−1.23, p=0.218).

### 3.6. Bayesian Weighted-Incidence Antibiogram

#### 3.6.1. Global Results

Among 68 patients with identified pathogens (64 bacterial, 4 fungal), 23 distinct bacterial species were identified. The Bayesian hierarchical model converged adequately (maximum $\hat{R}=1.005$, zero divergent transitions; Supplementary Figure S1 and Table S11). A total of 54 regimens (20 monotherapies, 34 combinations) were evaluated; complete Bayesian and non-Bayesian coverage estimates for all regimens are provided in Supplementary Table S9.

**Figure 5:**
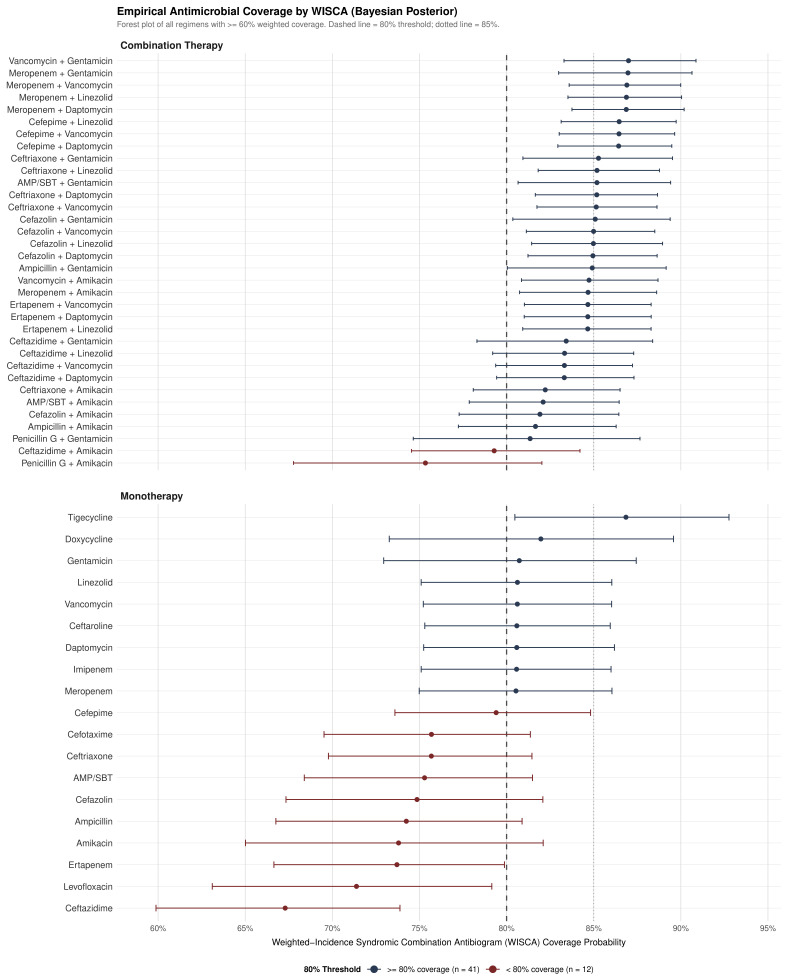
Bayesian weighted-incidence antibiogram: empirical antimicrobial coverage for infective endocarditis. Forest plot showing Bayesian posterior median coverage with 95% highest density intervals (HDI) for 53 of 54 regimens with ≥60% coverage (penicillin G excluded at 35%), stratified by monotherapy and combination therapy. The dashed vertical line indicates the 80% weighted coverage threshold; the dotted line indicates 85%. Blue: regimens meeting the ≥80% threshold (n=41); red: regimens below threshold (n=12). Complete coverage estimates for all 54 regimens are provided in Supplementary Table S9.

From an antimicrobial stewardship perspective, 41 of 54 regimens (76%) met or exceeded the 80% weighted coverage threshold (Figure 5). Among monotherapies, six agents achieved ≥80% Bayesian coverage: tigecycline (86.9%, HDI 80.5–92.8), doxycycline (82.0%), gentamicin (80.7%), vancomycin (80.6%), linezolid (80.6%), and daptomycin (80.6%). However, these coverage probabilities reflect microbiological susceptibility and do not account for pharmacokinetic considerations; notably, tigecycline and doxycycline achieve low serum concentrations due to their large volumes of distribution (>300 L), limiting their utility as initial empirical agents in endocarditis where sustained bactericidal serum levels are essential [32]. Moreover, pooled analyses have reported an excess mortality signal associated with tigecycline use in serious infections [33]. Vancomycin—the most commonly prescribed empirical agent for IE—met the 80% threshold, supporting its continued use as first-line empirical therapy. Combination regimens involving vancomycin or meropenem paired with an aminoglycoside achieved the highest coverage (vancomycin + gentamicin and meropenem + gentamicin, both 87.0%), though the incremental benefit over vancomycin monotherapy (6.4 percentage points) must be weighed against the nephrotoxicity risk of aminoglycosides, particularly in this hemodialysis-predominant population. Penicillin G (35.0%) and ceftazidime (67%) did not meet the 80% threshold, consistent with the predominance of staphylococci in our setting.

#### 3.6.2. Stratified Analysis by IE Type

**Figure 6:**
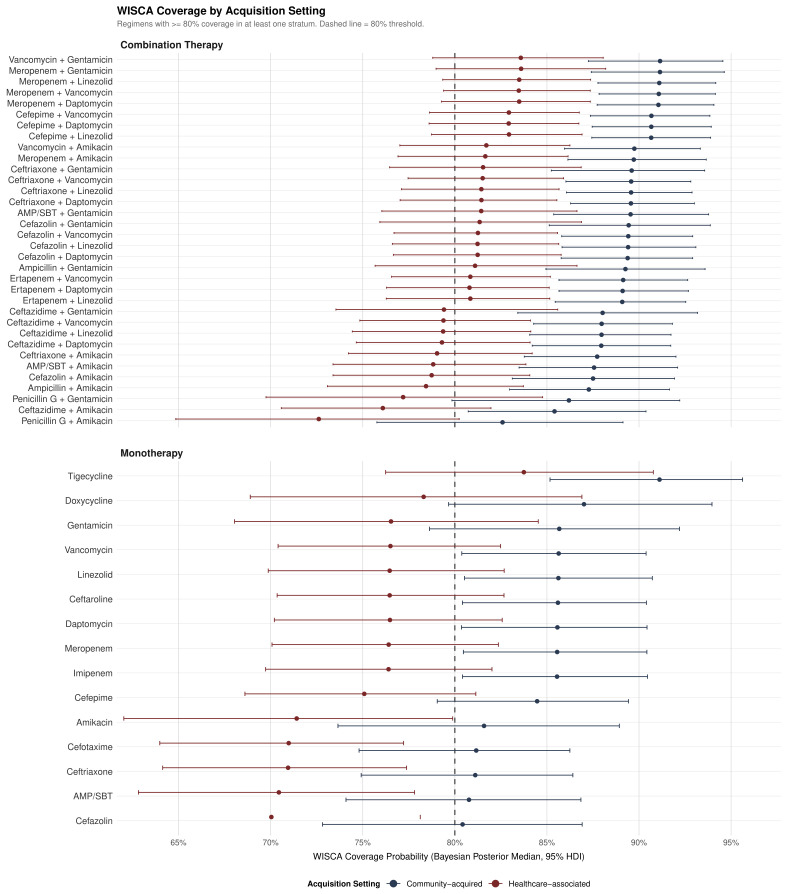
Bayesian WISCA coverage stratified by IE acquisition setting. Paired forest plot showing Bayesian posterior median coverage with 95% HDI for regimens meeting the ≥80% threshold in at least one stratum. Blue: community-acquired IE; red: healthcare-associated IE. The dashed vertical line indicates the 80% weighted coverage threshold. Community-acquired IE showed consistently higher coverage across all regimens, with a 7–9 percentage-point gap for top-ranked regimens.

Bayesian coverage was consistently higher for community-acquired IE across all 54 regimens (Figure 6; Supplementary Table S10). In community-acquired IE, narrower-spectrum combination regimens achieved adequate coverage: cephalosporin-based combinations including ceftriaxone + gentamicin, cefazolin + gentamicin, and ampicillin + gentamicin all exceeded the 80% threshold (range 85–90%), providing stewardship-compatible empirical options that avoid carbapenems and anti-MRSA agents. This is clinically relevant given that no methicillin-resistant *S. aureus* (MRSA) was identified in our cohort, and *Streptococcus* spp. dominated community-acquired isolates. Combinations with amikacin (ceftriaxone + amikacin, cefazolin + amikacin, ampicillin/sulbactam + amikacin) also exceeded 80%, offering aminoglycoside alternatives in settings where gentamicin resistance is a concern.

In healthcare-associated IE, vancomycin monotherapy fell below the 80% threshold (76.5%), suggesting that combination empirical therapy is more appropriate in this subpopulation. Cefazolin-based combinations (cefazolin + vancomycin, cefazolin + gentamicin, cefazolin + daptomycin) and ceftriaxone-based combinations (ceftriaxone + vancomycin, ceftriaxone + gentamicin) all exceeded 80% coverage (range 81–84%), providing reasonable alternatives to carbapenem-based regimens. Notably, ceftazidime + vancomycin and ceftazidime + gentamicin showed suboptimal healthcare-associated coverage (<80%), consistent with the limited gram-positive spectrum of ceftazidime. Despite the absolute coverage differences between strata (7–9 percentage-point gap), stratum-specific Bayesian coverages were nearly perfectly correlated (r=0.999), indicating that antimicrobial rankings were preserved across both settings.

## 4. Discussion

This study presents three main findings from a seven-year cohort of IE patients at a Mexican tertiary university hospital: (1) a parsimonious three-variable model demonstrated excellent discrimination for in-hospital mortality (AUC 0.922, optimism-corrected 0.908), significantly outperforming the RiskE and ICE international prognostic scores; (2) the Bayesian hierarchical antibiogram demonstrated that 41 of 54 evaluated regimens (76%) met the ≥80% coverage threshold, with narrower-spectrum cephalosporin + aminoglycoside combinations providing stewardship-compatible empirical options for community-acquired IE—supported by the complete absence of methicillin-resistant *S. aureus* (MRSA) in our cohort—while combination therapy was more appropriate for healthcare-associated IE where vancomycin monotherapy fell below the 80% threshold; and (3) the cohort exhibited a distinctive epidemiological phenotype—young, hemodialysis-predominant, with paradoxically low *S. aureus* mortality and no MRSA—that challenges assumptions underlying European-derived risk stratification tools.

### 4.1. Mortality and Epidemiological Context

The in-hospital mortality of 22.9% falls at the lower end of the range reported for Latin America (22–49%) and below the regional pooled estimate of 33% [2]. Several converging factors likely explain this favorable outcome. First, the surgical intervention rate of 63.2%, with 100% fulfillment of documented surgical indications, substantially exceeds the 28–40% reported in LMIC series [5] and approaches rates in high-income specialized centers. The survival benefit of early surgery in IE is well established [34], and high institutional adherence to surgical guidelines likely contributed to the relatively low mortality [35,36]. Second, the elevated proportion of right-sided IE (46.4%)—driven by the hemodialysis-predominant population—contributes favorably, as right-sided IE carries intrinsically lower mortality (8.9% in our cohort vs. 35.0% for left-sided) [37]. Third, the young age of the cohort (median 37 years vs. >60 years in European series) may confer a survival advantage.

The demographic profile of our cohort—young patients with healthcare-associated IE linked to hemodialysis vascular access—represents a distinct epidemiological phenotype that diverges from both the European/North American pattern (elderly patients with degenerative valves) and the classical LMIC pattern (young patients with rheumatic heart disease) [5,38]. This “hemodialysis-associated IE phenotype” has been increasingly recognized in Mexican centers [6] and likely reflects the high burden of chronic kidney disease in the region, combined with reliance on central venous catheters for hemodialysis access.

### 4.2. The S. aureus Mortality Paradox

A notable finding was the paradoxically low *S. aureus* mortality (12.9%) compared with international reports of 20–40% [39,40]. Stratified analysis revealed that 58% of *S. aureus* cases involved right-sided IE, which carries an inherently better prognosis regardless of the causative pathogen. Within left-sided IE, *S. aureus* mortality (18.2%) remained lower than non-*S. aureus* mortality (43.2%), possibly attributable to the high surgical rate in our institution. This finding has important methodological implications: the variable “high-risk pathogen” was excluded from the predictive model after demonstrating that its apparent prognostic effect was entirely confounded by IE laterality—a form of Simpson’s paradox [41]. This underscores the importance of careful stratified analysis before incorporating microbiological variables into predictive models in settings where pathogen distribution is strongly associated with IE laterality.

### 4.3. Failure of International Prognostic Scores

The poor discriminative performance of both RiskE (AUC 0.598) and ICE (AUC 0.632) in our cohort, with both scores statistically indistinguishable from chance, represents a clinically important finding. While these scores achieved AUCs of 0.76–0.82 in their derivation cohorts [7,8], their performance in external validations has been inconsistent. A meta-analysis of 12 prognostic scores found pooled AUCs ranging from 0.64 to 0.83 with substantial heterogeneity [10], and a systematic review concluded that no existing score comprehensively integrates clinical, microbiological, and echocardiographic data within the first 48–72 h [9].

Multiple factors explain the score failure in our population. First, RiskE was derived exclusively from patients with left-sided IE undergoing cardiac surgery in Spanish centers [7], whereas 46.4% of our patients had right-sided IE. Second, neither score incorporates hemodialysis, vascular access type, or healthcare-associated IE status—variables that define the risk profile of our population. Third, the concentrated score distributions (median RiskE 9 points in both survivors and non-survivors) reflect the inability of these tools to capture the principal axes of prognostic variability in this cohort. These findings argue against uncritical adoption of European-derived prognostic scores in Latin American settings and support the TRIPOD recommendation for local model development and validation [26].

### 4.4. Embolic Risk Prediction

The exploratory embolism model yielded several noteworthy findings. Valvular regurgitation was the strongest predictor of embolic events (Firth OR 5.02, p=0.001), consistent with evidence that hemodynamic turbulence from regurgitant flow may promote vegetation fragmentation and embolization [4,13]. Previous endocarditis was associated with reduced embolic risk (OR 0.16, p=0.034), a finding that may reflect earlier clinical recognition and more aggressive initial management in patients with known prior IE, or alternatively, a survivor bias whereby patients who survived a previous episode may represent a more resilient subpopulation [42].

The absence of vegetation characteristics (size > 10 mm, mobility) as significant predictors contrasts with the 2023 ESC guidelines, which emphasize vegetation size > 10 mm as a major criterion for surgical intervention to prevent embolism [13]. However, our finding aligns with recent evidence suggesting that the predictive value of vegetation size is attenuated in cohorts with high rates of *S. aureus* IE, where embolism occurs early and often before echocardiographic evaluation [43]. In our cohort, the high prevalence of *S. aureus* (32%) and the large median vegetation size (19 mm) may have reduced the discriminative capacity of vegetation metrics. The moderate AUC of 0.737 (corrected 0.719) and excellent calibration (Hosmer–Lemeshow p=0.936) suggest that the model captures meaningful risk variation, though the exploratory nature and wide confidence intervals warrant cautious interpretation.

### 4.5. Bayesian Weighted-Incidence Antibiogram

The WISCA analysis represents, to our knowledge, the first application of a Bayesian weighted-incidence antibiogram specifically for IE. The substantial bidirectional shrinkage between traditional and Bayesian estimates (r=0.683) highlights the value of hierarchical modeling in settings with sparse species-level susceptibility data. The low coverage of penicillin G (35.0%) reflects the predominance of staphylococci in our setting and argues against its use as empirical monotherapy.

From a stewardship perspective, the finding that multiple narrower-spectrum combination regimens—particularly cephalosporin + aminoglycoside combinations—exceeded the 80% coverage threshold in community-acquired IE is clinically actionable. The absence of MRSA in our cohort supports initial empirical regimens that do not require anti-MRSA coverage (vancomycin, linezolid, and daptomycin) for community-acquired presentations, reserving these agents for healthcare-associated IE where the resistance burden is higher. This approach aligns with current antimicrobial stewardship principles emphasizing de-escalation potential and avoidance of unnecessary broad-spectrum agents.

### 4.6. Limitations

This study has several limitations. First, its retrospective, single-center design introduces potential selection and information bias, and the results reflect the specific epidemiological profile of one institution. Second, the sample size (97 patients, 22 events) limits the number of predictors to three (EPV 7.3), and confidence intervals are wide. The reported AUC of 0.922 (corrected 0.908) warrants careful interpretation in this context. While the moderate EPV falls below the traditional rule of 10 [44], several lines of evidence mitigate overfitting concerns: bootstrap optimism was low (0.014), indicating stable discrimination; Firth penalization produced consistent estimates with narrower confidence intervals; LASSO independently confirmed the core predictors; the MICE sensitivity analysis yielded identical results (Table 5); and the calibration plot demonstrated adequate agreement between predicted and observed probabilities (Figure 3). Nevertheless, the wide confidence intervals for individual odds ratios (e.g., vasopressor-requiring shock OR 9.23, 95% CI 2.40–40.61) reflect the inherent imprecision of small-sample estimation, and the high AUC likely reflects, in part, the strong prognostic signal of vasopressor-requiring shock and acute heart failure rather than model complexity. Third, the absence of external validation is the principal limitation; the model’s performance in independent populations is unknown, and clinical adoption should await multicenter validation, consistent with TRIPOD Stage 2b recommendations [26]. Fourth, serum creatinine—a predictor in multiple international scores—was not available, which may have affected score calculations. Fifth, the 29.9% rate of culture-negative IE limits the WISCA sample size. Sixth, the generalizability of our findings is inherently limited by the distinctive epidemiological profile of this cohort (young age, 47.4% hemodialysis prevalence); the model should be considered a hypothesis-generating tool that is context-specific to this hemodialysis-predominant population rather than broadly applicable to all IE populations. Finally, the WISCA results apply to our institution’s microbial ecology and may not be generalizable to centers with different pathogen distributions.

### 4.7. Future Directions

Multicenter validation of the local predictive model across Mexican and Latin American hospitals is the immediate priority. Development of a regional prognostic score incorporating hemodialysis status and vascular access type could improve risk stratification in LMIC populations. Serial WISCA monitoring would enable tracking of temporal trends in antimicrobial coverage, and molecular characterization of culture-negative IE episodes could improve etiological precision.

## 5. Conclusions

In-hospital mortality from IE at this Mexican tertiary university hospital was 22.9%, at the lower end of the Latin American range. A parsimonious model comprising vasopressor-requiring shock (Firth OR 9.23), acute heart failure (OR 10.01), and left-sided IE (OR 2.66) achieved an AUC of 0.922 (optimism-corrected 0.908), significantly outperforming the RiskE (AUC 0.598) and ICE (AUC 0.632) international scores. The model requires external validation in independent cohorts before clinical adoption and should be considered context-specific to hemodialysis-predominant IE populations.

The Bayesian weighted-incidence antibiogram demonstrated that 41 of 54 regimens (76%) met the ≥80% coverage threshold. Vancomycin monotherapy (80.6%) represents a reasonable first-line empirical agent, while combination regimens offered incremental coverage (up to 87.0%) that must be balanced against toxicity risks. Coverage was consistently lower in healthcare-associated IE (7–9 percentage-point gap), suggesting that combination therapy may be more appropriate in this subpopulation; overall empirical therapy appropriateness was 83.6%. Narrower-spectrum cephalosporin + aminoglycoside combinations provide stewardship-compatible alternatives for community-acquired IE, while combination therapy is recommended for healthcare-associated IE.

Multicenter external validation of both the predictive model and the Bayesian antibiogram methodology in diverse Latin American IE cohorts is warranted.

## Figures and Tables

**Table 1:** Clinical characteristics stratified by in-hospital survival status.

Variable	Total (N=96) ^1^	Survivor (N=74) ^1^	Death (N=22) ^1^	*p* ^2^
Age (years), median (Q1, Q3)	37.5 (28.0, 52.5)	34.0 (25.0, 50.0)	50.5 (37.0, 66.0)	0.002
Sex, male	72 (75.0%)	57 (77.0%)	15 (68.2%)	0.411
BMI (kg/m^2^), median (Q1, Q3)	23.7 (21.5, 27.0)	22.9 (21.1, 25.7)	26.9 (23.8, 28.4)	0.006
Charlson index, median (Q1, Q3)	2.0 (0.5, 3.0)	2.0 (0.0, 2.0)	3.0 (2.0, 4.0)	0.010
Diabetes mellitus	24 (25.0%)	17 (23.0%)	7 (31.8%)	0.411
Chronic kidney disease	43 (44.8%)	35 (47.3%)	8 (36.4%)	0.466
Liver disease	2 (2.1%)	2 (2.7%)	0 (0.0%)	>0.999
Active neoplasm	1 (1.0%)	1 (1.4%)	0 (0.0%)	>0.999
HIV with immunosuppression	0 (0.0%)	0 (0.0%)	0 (0.0%)	>0.999
Intravenous drug use	3 (3.1%)	3 (4.1%)	0 (0.0%)	>0.999
Previous endocarditis	7 (7.3%)	5 (6.8%)	2 (9.1%)	0.658
Prosthetic valve	11 (11.5%)	8 (10.8%)	3 (13.6%)	0.710
CVC or Mahurkar catheter	44 (45.8%)	36 (48.6%)	8 (36.4%)	0.340
Cardiac electronic device	5 (5.3%)	5 (6.8%)	0 (0.0%)	0.583
Hemodialysis	46 (47.9%)	37 (50.0%)	9 (40.9%)	0.478
Recent hospitalization (3 mo)	43 (44.8%)	30 (40.5%)	13 (59.1%)	0.148
IE type				0.013
Community-acquired	42 (44.7%)	30 (41.1%)	12 (57.1%)	
Healthcare-associated	50 (53.2%)	43 (58.9%)	7 (33.3%)	
Nosocomial	2 (2.1%)	0 (0.0%)	2 (9.5%)	
Prosthetic vs. native IE				0.710
Native	85 (88.5%)	66 (89.2%)	19 (86.4%)	
Prosthetic	11 (11.5%)	8 (10.8%)	3 (13.6%)	
Affected side				0.003
Left	48 (50.0%)	30 (40.5%)	18 (81.8%)	
Right	45 (46.9%)	41 (55.4%)	4 (18.2%)	
Bilateral	2 (2.1%)	2 (2.7%)	0 (0.0%)	
Unclassified	1 (1.0%)	1 (1.4%)	0 (0.0%)	
Positive blood cultures	65 (71.4%)	49 (70.0%)	16 (76.2%)	0.784
Pathogen group				0.010
*Staphylococcus aureus*	31 (32.3%)	27 (36.5%)	4 (18.2%)	
*Streptococcus* spp.	12 (12.5%)	10 (13.5%)	2 (9.1%)	
*Enterococcus* spp.	3 (3.1%)	0 (0.0%)	3 (13.6%)	
Coagulase-negative staph.	8 (8.3%)	4 (5.4%)	4 (18.2%)	
Gram-negative organisms	9 (9.4%)	8 (10.8%)	1 (4.5%)	
Fungi	4 (4.2%)	2 (2.7%)	2 (9.1%)	
Culture-negative	29 (30.2%)	23 (31.1%)	6 (27.3%)	
LVEF (%), median (Q1, Q3)	60.0 (55.0, 63.0)	60.0 (54.0, 63.0)	59.5 (55.5, 64.0)	0.566
LVEF (category)				0.865
Preserved (≥50%)	80 (85.1%)	62 (83.8%)	18 (90.0%)	
Moderately reduced (40–49%)	10 (10.6%)	8 (10.8%)	2 (10.0%)	
Reduced (<40%)	4 (4.3%)	4 (5.4%)	0 (0.0%)	
Vegetation dimension (mm), median	19.0 (13.0, 24.0)	19.0 (15.0, 24.0)	17.0 (12.0, 28.0)	0.601
Vegetation > 10 mm	69 (85.2%)	53 (84.1%)	16 (88.9%)	>0.999
Number of vegetations				0.424
1	62 (76.5%)	46 (73.0%)	16 (88.9%)	
2	17 (21.0%)	15 (23.8%)	2 (11.1%)	
3	2 (2.5%)	2 (3.2%)	0 (0.0%)	
Embolism	43 (44.8%)	30 (40.5%)	13 (59.1%)	0.148
Acute heart failure	26 (27.1%)	9 (12.2%)	17 (77.3%)	<0.001
Vasopressor-requiring shock	19 (19.8%)	6 (8.1%)	13 (59.1%)	<0.001
Arrhythmias	2 (2.1%)	0 (0.0%)	2 (9.1%)	0.051
ICU admission	18 (18.8%)	9 (12.2%)	9 (40.9%)	0.005
Total complications, median (Q1, Q3)	2.0 (1.0, 3.0)	2.0 (0.0, 3.0)	4.0 (2.0, 5.0)	<0.001
SOFA-2 score, median (Q1, Q3)	3.0 (0.0, 4.0)	4.0 (0.0, 4.0)	3.0 (1.0, 4.0)	0.556
RiskE score (points), median (Q1, Q3)	9.0 (0.0, 13.0)	9.0 (0.0, 9.0)	9.0 (7.0, 14.0)	0.161
ICE score (points), median (Q1, Q3)	7.0 (5.0, 9.0)	7.0 (5.0, 9.0)	9.0 (6.0, 11.0)	0.064
Surgical indication	59 (74.7%)	39 (66.1%)	20 (100.0%)	0.002
Surgery performed	60 (63.2%)	49 (67.1%)	11 (50.0%)	0.207
Surgical indication fulfilled	59 (63.4%)	48 (67.6%)	11 (50.0%)	0.204
Hospital stay (days), median (Q1, Q3)	35.0 (25.0, 51.0)	34.0 (26.0, 50.0)	36.0 (19.0, 52.0)	0.930

^1^ *n* (%) or median (Q1, Q3). ^2^ Wilcoxon rank-sum test for continuous variables; chi-squared or Fisher’s exact test for categorical variables. BMI: body mass index; CVC: central venous catheter; IE: infective endocarditis; LVEF: left ventricular ejection fraction.

**Table 2:** Bivariate screening of candidate predictors for in-hospital mortality, organized by 16 clinical domains (n=96, 22 events). Crude odds ratios with 95% confidence intervals from univariate logistic regression. Multilevel categorical variables: global *p*-value from likelihood ratio test. NE: not estimable due to quasi-complete separation. Sixty-two variables screened in two phases.

Domain	Vars. Screened	Notable Significant Associations (p<0.05)	Candidates (p<0.20)
Demographics	4	Age per year (OR 1.04, 95% CI 1.01–1.08, p=0.005); Charlson per point (OR 1.30, 95% CI 1.02–1.66, p=0.032)	2
Comorbidities	6	None	0
Risk factors	5	None	1
IE classification	4	Affected side (LRT p=0.005); IE type (LRT p=0.011)	3
Microbiology	2	Pathogen group (LRT p=0.013)	1
Echocardiography	5	Total complications per unit (OR 2.20, 95% CI 1.53–3.40, p<0.001)	1
Complications	5	Vasopressor-requiring shock (OR 16.37, 95% CI 5.21–57.90, p<0.001); acute heart failure (OR 8.67, 95% CI 2.98–26.86, p<0.001); ICU admission (OR 5.00, 95% CI 1.67–15.35, p=0.004)	4
Treatment	3	None	2
Prognostic scores	3	ICE per point (OR 1.20, 95% CI 1.04–1.42, p=0.017)	3
Derived variables	8	Left-sided IE (OR 3.24, 95% CI 1.14–9.86, p=0.030); high-risk pathogen (p=0.001); structural complication (p=0.002)	4
Individual complications	7	Aneurysm (p=0.013); valvular compromise (p=0.039)	3
Clinical presentation	4	Vascular/immunological phenomena (p=0.011)	1
Biomarkers	2	None	0
Temporal variables	3	None	0
Treatment/microbiology	4	None	0
Additional risk factors	7	None	0
Total	62		25

**Table 3:** Adjusted odds ratios by model.

Model	Predictor	OR (95% CI)	*p*
Model 1: Backward AIC (4 vars)	Age (years)	1.05 (1.00–1.11)	0.049
	Vasopressor-requiring shock	4.29 (0.91–22.98)	0.072
	Acute heart failure	45.58 (8.42–462.31)	0.0001
	Surgery	0.28 (0.04–1.60)	0.173
Model 2: Final (3 vars)	Vasopressor-requiring shock	11.14 (2.67–55.52)	0.002
	Left-sided IE	2.93 (0.62–15.92)	0.182
	Acute heart failure	12.11 (3.12–55.72)	0.0006
Model 2b: Firth (3 vars)	Vasopressor-requiring shock	9.23 (2.40–40.61)	0.001
	Left-sided IE	2.66 (0.62–12.68)	0.185
	Acute heart failure	10.01 (2.78–41.07)	0.0004
Model D: 30-day mortality	Vasopressor-requiring shock	9.14 (2.07–47.90)	0.005
	Left-sided IE	3.38 (0.83–15.36)	0.094
	Acute heart failure	7.31 (1.91–30.15)	0.004
Model E: Embolism	Valvular regurgitation	5.38 (2.08–15.12)	0.001
	Prior antibiotic use	0.40 (0.11–1.28)	0.135
	Previous endocarditis	0.11 (0.01–0.77)	0.055

OR, odds ratio; CI, confidence interval; IE, infective endocarditis; EPV, events per variable. Model 1, backward AIC from Phase 1 candidates (4 predictors; EPV = 5.5). Model 2 (final), clinical model from Phase 2 extended screening (3 predictors; EPV = 7.3). Model 2b, Firth-penalized estimates for the same 3 predictors. Model D, the same 3 predictors applied to 30-day all-cause mortality (n=86; 24 events; EPV = 8.0). Model E, embolism model derived after univariate screening and backward AIC (complete-case n=93; 42 events; EPV = 14.0); Model E-Firth, Firth-penalized estimates for the same predictors. A comprehensive multi-model comparison, including all fitted models, LASSO specifications, and international scores is provided in Supplementary Table S8.

**Table 4:** Multivariable logistic regression model for in-hospital mortality (n=96, 22 events).

Predictor	GLM OR (95% CI)	*p*	Firth OR (95% CI)	Firth *p*
Vasopressor-requiring shock	11.14 (2.67–55.52)	0.001	9.23 (2.40–40.61)	0.001
Left-sided IE	2.93 (0.62–15.92)	0.182	2.66 (0.62–12.68)	0.185
Acute heart failure	12.11 (3.12–55.72)	<0.001	10.01 (2.78–41.07)	<0.001

Model performance: AUC 0.922 (95% CI 0.87–0.98); optimism-corrected AUC 0.908 (optimism 0.014, B=1000); AIC 64.8; Hosmer–Lemeshow p=0.505; Nagelkerke $R^{2}=0.583$; Brier score 0.092 (trivial 0.177).

**Table 5:** Sensitivity analysis: complete-case analysis vs. multiple imputation (MICE, m=20, CART method).

Predictor	CCA OR (95% CI)	MICE OR (95% CI)	CCA *p*	MICE *p*
Vasopressor-requiring shock	11.14 (2.67–55.52)	11.12 (2.47–50.11)	0.001	0.001
Left-sided IE	2.93 (0.62–15.92)	2.87 (0.58–14.23)	0.182	0.193
Acute heart failure	12.11 (3.12–55.72)	12.28 (2.92–51.60)	<0.001	<0.001
AUC (95% CI)	0.922 (0.87–0.98)	0.922 (0.920–0.925) ^a^		

^a^ Mean AUC across 20 imputed datasets (range). CCA: complete-case analysis; MICE: multiple imputation by chained equations; CART: classification and regression trees.

**Table 6:** Comparison of discriminative performance: local model vs. international scores.

Comparison	AUC 1	AUC 2	ΔAUC	DeLong *z*	*p*
Model 2 vs. Model 1	0.922	0.933	−0.011	−0.86	0.388
Model 2 vs. RiskE	0.922	0.598	0.324	4.69	<0.0001
Model 2 vs. ICE	0.922	0.632	0.290	4.08	<0.0001
Model 1 vs. RiskE	0.933	0.598	0.335	5.10	<0.0001
Model 1 vs. ICE	0.933	0.632	0.301	4.52	<0.0001
RiskE vs. ICE	0.598	0.632	−0.034	−0.55	0.581

AUCs for comparisons involving international scores are computed on the paired subset (n=95) with available scores; full-cohort AUC for Model 2 is 0.922 (n=96). Model 2 AUC on the paired subset is 0.933.

**Table 7:** Exploratory logistic regression model for embolic events (n=93, 42 events).

Predictor	GLM OR (95% CI)	*p*	Firth OR (95% CI)	Firth *p*
Valvular regurgitation	5.38 (2.08–15.12)	0.001	5.02 (1.98–13.72)	0.001
Prior antibiotic use	0.40 (0.11–1.28)	0.135	0.43 (0.13–1.31)	0.140
Previous endocarditis	0.11 (0.01–0.77)	0.055	0.16 (0.02–0.88)	0.034

Model performance: AUC 0.737 (95% CI 0.64–0.83); optimism-corrected AUC 0.719 (optimism 0.017, B=1000); EPV 14.0; Hosmer–Lemeshow p=0.936.

## Data Availability

The datasets analyzed during the current study are available from the corresponding author upon reasonable request.

## Supplementary Materials

The following supporting information can be downloaded at: https://www.mdpi.com/article/10.3390/medsci14020214/s1, Figure S1: MCMC convergence diagnostics for the Bayesian WISCA models; Table S1: Clinical characteristics by IE type; Table S2: Clinical characteristics by ICU admission; Table S3: Clinical characteristics by prolonged hospital stay; Table S4: Clinical characteristics by complicated IE; Table S5: Multivariable models for secondary outcomes; Table S6: Empirical antimicrobial regimens; Table S7: Extended univariate analysis; Table S8: Multi-model comparison; Table S9: Combined global WISCA coverage; Table S10: Stratified WISCA by IE type; Table S11: WISCA convergence diagnostics.

## Abbreviations

AUC	Area Under the Curve
BMI	Body Mass Index
CCA	Complete-Case Analysis
CI	Confidence Interval
DCA	Decision Curve Analysis
EPV	Events per Variable
ESC	European Society of Cardiology
HDI	Highest Density Interval
ICE	International Collaboration on Endocarditis score
ICU	Intensive Care Unit
IDI	Integrated Discrimination Improvement
IE	Infective Endocarditis
IQR	Interquartile Range
LASSO	Least Absolute Shrinkage and Selection Operator
LMIC	Low- and Middle-Income Country
LVEF	Left Ventricular Ejection Fraction
MCMC	Markov Chain Monte Carlo
MCAR	Missing Completely at Random
MICE	Multiple Imputation by Chained Equations
MRSA	Methicillin-Resistant *Staphylococcus aureus*
NRI	Net Reclassification Improvement
OR	Odds Ratio
RiskE	Risk-Endocarditis score
ROC	Receiver Operating Characteristic
SOFA-2	Sequential Organ Failure Assessment-2
VIF	Variance Inflation Factor
WISCA	Weighted-Incidence Syndromic Combination Antibiogram

## References

[1] Global Burden of Cardiovascular Diseases and Risks 2023 Collaborators (2025). Global, regional, and national burden of cardiovascular diseases and risk factors in 204 countries and territories, 1990–2023. J. Am. Coll. Cardiol..

[2] Tzoumas A., Sagris M., Xenos D., Ntoumaziou A., Kyriakoulis I., Kakargias F., Liaqat W., Nagraj S., Patel R., Korosoglou G. (2025). Epidemiological profile and mortality of infective endocarditis over the past decade: A systematic review and meta-analysis of 133 studies. Am. J. Cardiol..

[3] Feng J., Liu P., Li H., Chen H., Shen Q., Liu H., Hu J. (2025). Global, regional, and national burden of endocarditis, 1990–2021: A systematic analysis of the GBD 2021 study. BMC Cardiovasc. Disord..

[4] Li M., Kim J.B., Sastry B.K.S., Chen M. (2024). Infective endocarditis. Lancet.

[5] Mutagaywa R.K., Vroon J.C., Fundikira L., Wind A.M., Kunambi P., Manyahi J., Kamuhabwa A., Kwesigabo G., Chamuleau S.A., Cramer M.J. (2022). Infective endocarditis in developing countries: An update. Front. Cardiovasc. Med..

[6] Ruiz-Beltran A.M., Barron-Magdaleno C., Ruiz-Beltran S.M., Sánchez-Villa J.D., Orihuela-Sandoval C., Oseguera-Moguel J., Payro-Ramírez G. (2022). Infective endocarditis: 10-year experience in a non-cardiovascular center. Arch. Cardiol. Méx..

[7] Olmos C., Vilacosta I., Habib G., Maroto L., Fernández C., López J., Sarriá C., Salaun E., Di Stefano S., Carnero M. (2017). Risk score for cardiac surgery in active left-sided infective endocarditis. Heart.

[8] Park L.P., Chu V.H., Peterson G., Skoutelis A., Lejko-Zupa T., Bouza E., Tattevin P., Habib G., Tan R., Gonzalez J. (2016). Validated risk score for predicting 6-month mortality in infective endocarditis. J. Am. Heart Assoc..

[9] Rizzo V., Salmasi M.Y., Sabetai M., Primus C., Sandoe J., Lewis M., Woldman S., Athanasiou T. (2023). Infective endocarditis: Do we have an effective risk score model? A systematic review. Front. Cardiovasc. Med..

[10] Agrawal A., Arockiam A.D., Dahdah J.E., Honnekeri B., Schleicher M., Shekhar S., Haroun E., Witten J., Majid M., Pettersson G. (2026). Comparisons of risk scores for infective endocarditis surgery: A meta-analysis. Angiology.

[11] Mikus E., Sangiorgi D., Calvi S., Fiorentino M., Tenti E., Dalle Mura F., Savini C. (2025). Enhanced risk stratification in infective endocarditis surgery: A comprehensive external validation of all available mortality prediction scores. Clin. Epidemiol..

[12] Urina-Jassir M., Jaimes-Reyes M.A., Martinez-Vernaza S., Quiroga-Vergara C., Urina-Triana M. (2022). Clinical, microbiological, and imaging characteristics of infective endocarditis in Latin America: A systematic review. Int. J. Infect. Dis..

[13] Delgado V., Ajmone Marsan N., de Waha S., Bonaros N., Brida M., Burri H., Caselli S., Doenst T., Ederhy S., Erba P.A. (2023). 2023 ESC Guidelines for the management of endocarditis. Eur. Heart J..

[14] Peláez Ballesta A.I., García Vázquez E., Gómez Gómez J. (2022). Infective endocarditis treated in a secondary hospital: Epidemiological, clinical, microbiological characteristics and prognosis. Rev. Esp. Quimioter..

[15] Hebert C., Ridgway J., Vekhter B., Brown E.C., Weber S.G., Robicsek A. (2012). Demonstration of the weighted-incidence syndromic combination antibiogram: An empiric prescribing decision aid. Infect. Control Hosp. Epidemiol..

[16] Briseno-Ramírez J., Gómez-Quiroz A., Avila-Cardenas B.B., De Arcos-Jiménez J.C., Perales-Guerrero L., Andrade-Villanueva J.F., Martínez-Ayala P. (2025). Development of a weighted-incidence syndromic combination antibiogram (WISCA) to guide empiric antibiotic treatment for ventilator-associated pneumonia in a Mexican tertiary care university hospital. BMC Infect. Dis..

[17] Gómez-Quiroz A., Avila-Cardenas B.B., De Arcos-Jiménez J.C., Perales-Guerrero L., Martínez-Ayala P., Briseno-Ramirez J. (2025). The clinical implications of inappropriate therapy in community-onset urinary tract infections and the development of a Bayesian hierarchical weighted-incidence syndromic combination antibiogram. Antibiotics.

[18] Indrawati L., Sugianli A.K., Prakoso B.J., Gunawan A., Soerarso R., Soesanto A.M., Dewi A.D. (2024). Antibiotic susceptibility among infective endocarditis population: Syndromic antibiogram evaluation at Indonesian National Cardiovascular Center. Indones. J. Clin. Pathol. Med. Lab..

[19] Barbieri E., Bottigliengo D., Tellini M., Minotti C., Marchiori M., Cavicchioli P., Gregori D., Giaquinto C., Da Dalt L., Donà D. (2021). Development of a weighted-incidence syndromic combination antibiogram to guide the choice of the empiric antibiotic treatment for urinary tract infection in paediatric patients: A Bayesian approach. Antimicrob. Resist. Infect. Control.

[20] Liberati C., Donà D., Maestri L., Petris M.G., Barbieri E., Gallo E., Gallocchio J., Pierobon M., Calore E., Zin A. (2024). Application of the weighted-incidence syndromic combination antibiogram (WISCA) to guide the empiric antibiotic treatment of febrile neutropenia in oncological paediatric patients. Ann. Clin. Microbiol. Antimicrob..

[21] Salm J., Ikker F., Noory E., Beschorner U., Kramer T.S., Westermann D., Zeller T. (2024). Weighted-incidence syndromic combination antibiogram (WISCA) to support empirical antibiotic therapy decisions in infected ischemic leg ulcers—A feasibility study. J. Clin. Med..

[22] Li J.S., Sexton D.J., Mick N., Nettles R., Fowler V.G., Ryan T., Bashore T., Corey G.R. (2000). Proposed modifications to the Duke criteria for the diagnosis of infective endocarditis. Clin. Infect. Dis..

[23] Ranzani O.T., Singer M., Salluh J.I.F., Shankar-Hari M., Pilcher D., Berger-Estilita J., Coopersmith C.M., Juffermans N.P., Laffey J., Reinikainen M. (2025). Development and validation of the Sequential Organ Failure Assessment (SOFA)-2 score. JAMA.

[24] Steyerberg E.W. (2019). Clinical Prediction Models.

[25] Harrell F.E. (2015). Regression Modeling Strategies.

[26] Collins G.S., Reitsma J.B., Altman D.G., Moons K.G.M. (2015). Transparent Reporting of a multivariable prediction model for Individual Prognosis or Diagnosis (TRIPOD). Ann. Intern. Med..

[27] Firth D. (1993). Bias reduction of maximum likelihood estimates. Biometrika.

[28] DeLong E.R., DeLong D.M., Clarke-Pearson D.L. (1988). Comparing the areas under two or more correlated receiver operating characteristic curves. Biometrics.

[29] Pencina M.J., D’Agostino R.B., Steyerberg E.W. (2011). Extensions of net reclassification improvement calculations to measure usefulness of new biomarkers. Stat. Med..

[30] CLSI (2024). M39: Analysis and Presentation of Cumulative Antimicrobial Susceptibility Test Data.

[31] Bürkner P.-C. (2017). brms: An R package for Bayesian multilevel models using Stan. J. Stat. Softw..

[32] Rubino C.M., Bhavnani S.M., Forrest A., Dukart G., Dartois N., Cooper A., Korth-Bradley J., Ambrose P.G. (2012). Pharmacokinetics-pharmacodynamics of tigecycline in patients with community-acquired pneumonia. Antimicrob. Agents Chemother..

[33] Prasad P., Sun J., Danner R.L., Natanson C. (2012). Excess deaths associated with tigecycline after approval based on noninferiority trials. Clin. Infect. Dis..

[34] Kang D.H., Kim Y.J., Kim S.H., Sun B.J., Kim D.H., Yun S.C., Song J.M., Choo S.J., Chung C.H., Song J.K. (2012). Early surgery versus conventional treatment for infective endocarditis. N. Engl. J. Med..

[35] Mejia O.A.V., de Mendonça F.C.C., Sampaio L.A.B.N., Galas F.R.B.G., Pontes M.F., Caneo L.F., Dallan L.R.P., Lisboa L.A.F., Ferreira J.F.M., Dallan L.A.O. (2022). Adherence to the cardiac surgery checklist decreased mortality at a teaching hospital: A retrospective cohort study. Clinics.

[36] Chirillo F., Scotton P., Rocco F., Rigoli R., Borsatto F., Pedrocco A., De Leo A., Minniti G., Polesel E., Olivari Z. (2013). Impact of a multidisciplinary management strategy on the outcome of patients with native valve infective endocarditis. Am. J. Cardiol..

[37] Chahoud J., Sharif Yakan A., Saad H., Kanj S.S. (2016). Right-sided infective endocarditis and pulmonary infiltrates: An update. Cardiol. Rev..

[38] Santos D.A.M., Siciliano R.F., Besen B.A.M.P., Strabelli T.M.V., Sambo C.T., Milczwski V.M., Goldemberg F., Tarasoutchi F., Vieira M.L.C., Paixão M.R. (2024). Changing trends in clinical characteristics and in-hospital mortality of patients with infective endocarditis over four decades. J. Infect. Public Health.

[39] Elderia A., Hinzmann J., Soehne P., Bennour W., Wahlers T., Weber C. (2025). Surgery in *Staphylococcus aureus* infective endocarditis. J. Clin. Med..

[40] Østergaard L., Voldstedlund M., Bruun N.E., Bundgaard H., Iversen K., Køber N., Dahl A., Chamat-Hedem S., Petersen J.K., Jensen A.D. (2022). Prevalence and mortality of infective endocarditis in community-acquired and healthcare-associated *Staphylococcus aureus* bacteremia. Open Forum Infect. Dis..

[41] Blyth C.R. (1972). On Simpson’s paradox and the sure-thing principle. J. Am. Stat. Assoc..

[42] Miller D.P., Gomberg-Maitland M., Humbert M. (2012). Survivor bias and risk assessment. Eur. Respir. J..

[43] Murdoch D.R., Corey G.R., Hoen B., Miró J.M., Fowler V.G., Bayer A.S., Karchmer A.W., Olaison L., Pappas P.A., Moreillon P. (2009). Clinical presentation, etiology, and outcome of infective endocarditis in the 21st century: The ICE-PCS. Arch. Intern. Med..

[44] Vittinghoff E., McCulloch C.E. (2007). Relaxing the rule of ten events per variable in logistic and Cox regression. Am. J. Epidemiol..

