# Supplementary material for: In-Hospital Mortality Predictors and a Bayesian Weighted-Incidence Antibiogram in Infective Endocarditis: A Seven-Year Cohort Study from a Mexican Tertiary University Hospital
Source: Med Sci (Basel). 2026 Apr 26;14(2):214. doi: 10.3390/medsci14020214 (PMC13214617; doi:10.3390/medsci14020214)
Supplement: Supplementary file 1 [file medsci-14-00214-s001.zip › medsci-4186218-supplementary.pdf]

Supplementary Material

In-Hospital Mortality Predictors and a Bayesian Weighted-Incidence Antibiogram in Infective Endocarditis: A Seven-Year Cohort Study from a Mexican Tertiary University Hospital

Contents

Figure S1: MCMC Convergence Diagnostics **2**

Supplementary Tables S1–S11 **3**

Table S1: IE Type Stratification 4
Table S2: ICU Admission 6
Table S3: Prolonged Hospital Stay 8
Table S4: Complicated IE 10
Table S5: Multivariable Models for Secondary Outcomes 12
Table S6: Empirical Antimicrobial Regimens 13
Table S7: Extended Univariate Analysis 14
Table S8: Multi-Model Comparison 16
Table S9: Global WISCA Coverage 17
Table S10: Stratified WISCA by IE Type 19
Table S11: WISCA Convergence Diagnostics 21

Supplementary Figure S1

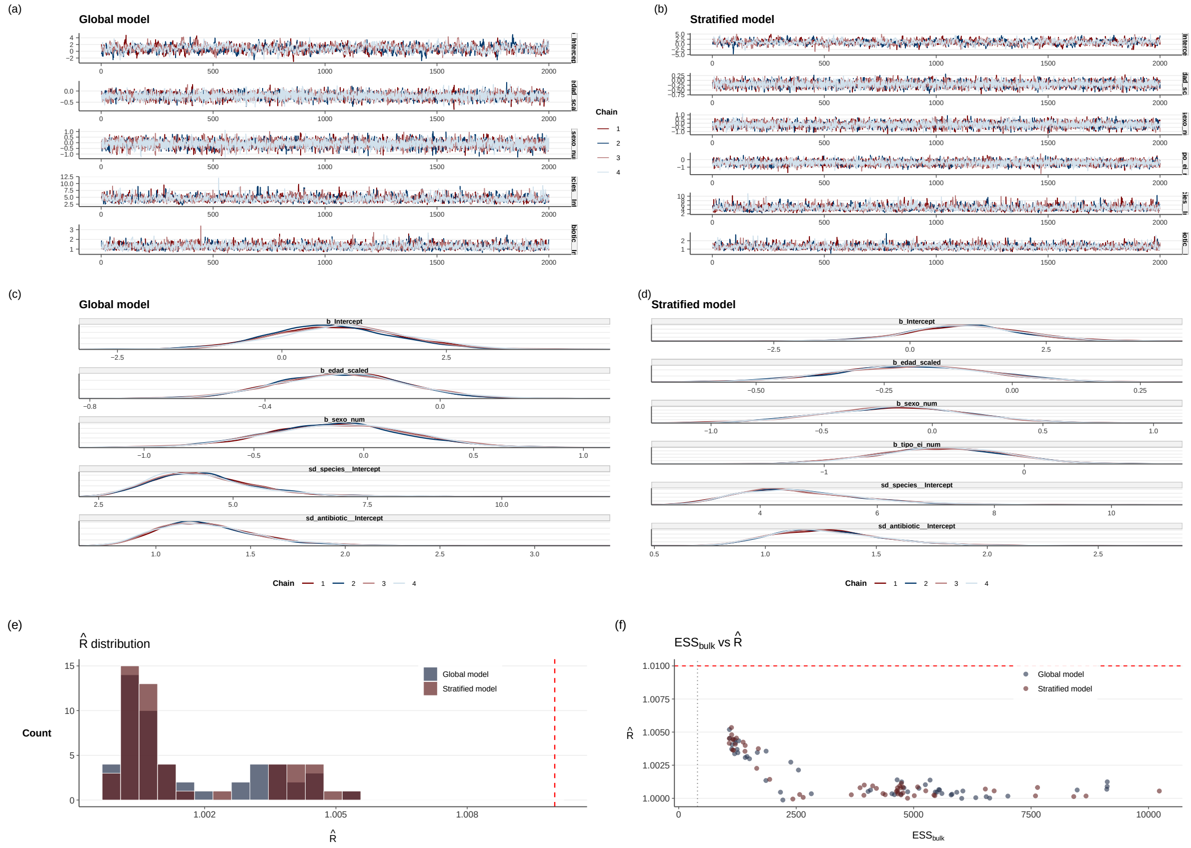

Figure 1: **Markov chain Monte Carlo convergence diagnostics for the Bayesian weighted-incidence antibiogram models.** (**a**,**b**) Trace plots for global and stratified models showing adequate mixing across four chains. (**c**,**d**) Posterior density overlays by chain demonstrating convergence. (**e**) Distribution of $\hat{R}$ values across all parameters (all < 1.01). (**f**) Effective sample size (ESS bulk) vs. $\hat{R}$ scatter plot confirming adequate sampling efficiency.

Supplementary Tables

Table S1: Clinical Characteristics Stratified by IE Type (Community-Acquired vs. Healthcare-Associated)

Variable	Total (N=94)	Community-acquired N = 42	Healthcare-associated N = 52	p-value
**Age (years)**				0.555
Median (Q1, Q3)	37.5 (28.0, 53.0)	45.0 (25.0, 53.0)	36.0 (29.0, 52.5)	
Mean (SD)	41.6 (16.6)	42.9 (17.3)	40.7 (16.1)	
**Sex**				0.002
Male	70 (74.5%)	38 (90.5%)	32 (61.5%)	
Female	24 (25.5%)	4 (9.5%)	20 (38.5%)	
**BMI (kg/m2)**				0.675
Median (Q1, Q3)	23.5 (21.2, 26.2)	23.5 (21.5, 27.0)	23.5 (21.1, 25.6)	
Mean (SD)	23.7 (3.8)	23.9 (4.1)	23.7 (3.5)	
Missing	12	8	4	
**Charlson Comorbidity Index**				<0.001
Median (Q1, Q3)	2.0 (0.0, 3.0)	1.0 (0.0, 2.0)	2.0 (2.0, 3.5)	
Mean (SD)	2.1 (2.0)	1.4 (1.8)	2.7 (1.9)	
**Diabetes mellitus**	23 (24.5%)	10 (23.8%)	13 (25.0%)	>0.999
**Chronic kidney disease**	42 (44.7%)	1 (2.4%)	41 (78.8%)	<0.001
**Liver disease**	2 (2.1%)	1 (2.4%)	1 (1.9%)	>0.999
**Active neoplasm**	1 (1.1%)	1 (2.4%)	0 (0.0%)	0.447
**Intravenous drug use**	3 (3.2%)	1 (2.4%)	2 (3.8%)	>0.999
**Previous endocarditis**	7 (7.4%)	2 (4.8%)	5 (9.6%)	0.455
**Prosthetic valve**	11 (11.7%)	3 (7.1%)	8 (15.4%)	0.335
**CVC or Mahurkar catheter**	43 (45.7%)	0 (0.0%)	43 (82.7%)	<0.001
**Cardiac electronic device**	5 (5.4%)	3 (7.1%)	2 (3.9%)	0.655
Missing	1	0	1	
**Hemodialysis**	45 (47.9%)	0 (0.0%)	45 (86.5%)	<0.001
**Recent hospitalization (3 months)**	42 (44.7%)	13 (31.0%)	29 (55.8%)	0.022
**Prosthetic vs. native IE**				0.335
Native	83 (88.3%)	39 (92.9%)	44 (84.6%)	
Prosthetic	11 (11.7%)	3 (7.1%)	8 (15.4%)	
**Affected side**				<0.001
Left	46 (48.9%)	33 (78.6%)	13 (25.0%)	
Right	45 (47.9%)	8 (19.0%)	37 (71.2%)	
Bilateral	2 (2.1%)	1 (2.4%)	1 (1.9%)	
Unclassified	1 (1.1%)	0 (0.0%)	1 (1.9%)	
**Positive blood cultures**	64 (71.9%)	24 (60.0%)	40 (81.6%)	0.033
Missing	5	2	3	
**Pathogen group**				<0.001
Staphylococcus aureus	31 (33.0%)	7 (16.7%)	24 (46.2%)	
Streptococcus spp.	12 (12.8%)	11 (26.2%)	1 (1.9%)	
Enterococcus spp.	3 (3.2%)	2 (4.8%)	1 (1.9%)	
Coagulase-negative staphylococci	7 (7.4%)	3 (7.1%)	4 (7.7%)	
Gram-negative organisms	9 (9.6%)	1 (2.4%)	8 (15.4%)	
Fungi	4 (4.3%)	1 (2.4%)	3 (5.8%)	
Culture-negative	28 (29.8%)	17 (40.5%)	11 (21.2%)	
**LVEF (%)**				0.792
Median (Q1, Q3)	60.0 (55.0, 63.0)	60.0 (55.0, 61.0)	59.0 (55.0, 64.0)	
Mean (SD)	57.5 (8.7)	56.8 (8.9)	58.1 (8.5)	

Continued on next page

Table S1 continued

Variable	Total (N=94)	Community-acquired N = 42	Healthcare-associated N = 52	p-value
Missing	2	1	1	
**LVEF (category)**				0.596
Preserved (≥50%)	78 (84.8%)	33 (80.5%)	45 (88.2%)	
Moderately reduced (40-49%)	10 (10.9%)	6 (14.6%)	4 (7.8%)	
Reduced (<40%)	4 (4.3%)	2 (4.9%)	2 (3.9%)	
Missing	2	1	1	
**Maximum vegetation dimension (mm)**				0.076
Median (Q1, Q3)	19.0 (12.8, 24.0)	17.0 (12.0, 21.0)	20.0 (15.0, 28.0)	
Mean (SD)	20.1 (9.7)	17.9 (7.6)	21.8 (10.8)	
Missing	14	7	7	
**Vegetation >10 mm**	68 (85.0%)	29 (82.9%)	39 (86.7%)	0.755
Missing	14	7	7	
**Embolism**	42 (44.7%)	25 (59.5%)	17 (32.7%)	0.012
**Acute heart failure**	24 (25.5%)	14 (33.3%)	10 (19.2%)	0.155
**Vasopressor-requiring shock**	19 (20.2%)	8 (19.0%)	11 (21.2%)	>0.999
**Arrhythmias**	2 (2.1%)	1 (2.4%)	1 (1.9%)	>0.999
**ICU admission**	18 (19.1%)	9 (21.4%)	9 (17.3%)	0.793
**Total complications**				0.001
Median (Q1, Q3)	2.0 (1.0, 3.0)	3.0 (2.0, 4.0)	1.5 (0.0, 3.0)	
Mean (SD)	2.1 (1.6)	2.7 (1.6)	1.6 (1.5)	
**SOFA-2 score**				<0.001
Median (Q1, Q3)	3.5 (1.0, 4.0)	1.0 (0.0, 2.0)	4.0 (4.0, 4.0)	
Mean (SD)	2.7 (2.3)	1.5 (2.1)	3.7 (2.0)	
**RiskE score (points)**				0.078
Median (Q1, Q3)	9.0 (0.0, 13.0)	7.0 (0.0, 9.0)	9.0 (4.5, 14.0)	
Mean (SD)	9.1 (8.4)	7.4 (6.9)	10.4 (9.3)	
Missing	1	1	0	
**ICE score (points)**				<0.001
Median (Q1, Q3)	7.0 (5.0, 9.0)	5.0 (4.0, 7.0)	8.0 (7.0, 10.0)	
Mean (SD)	7.2 (3.4)	5.5 (3.6)	8.6 (2.5)	
Missing	1	1	0	
**Surgical indication**	57 (74.0%)	31 (93.9%)	26 (59.1%)	<0.001
Missing	17	9	8	
**Surgery performed**	58 (62.4%)	33 (78.6%)	25 (49.0%)	0.005
Missing	1	0	1	
**Hospital stay (days)**				0.991
Median (Q1, Q3)	35.0 (26.0, 51.0)	35.5 (28.0, 47.0)	31.0 (21.0, 60.0)	
Mean (SD)	40.6 (25.3)	38.4 (20.5)	42.5 (28.6)	
Missing	1	0	1	
**In-hospital mortality**				0.220
Survivor	73 (77.7%)	30 (71.4%)	43 (82.7%)	
Death	21 (22.3%)	12 (28.6%)	9 (17.3%)	

[1]n (%)

[2]Wilcoxon rank sum test; Fisher's exact test

Table S2: Clinical Characteristics by ICU Admission

Variable	Total (N=96)	No N = 78	Yes N = 18	p-value
**Age (years)**				0.746
Median (Q1, Q3)	37.5 (28.0, 52.5)	36.5 (28.0, 52.0)	46.5 (25.0, 53.0)	
Mean (SD)	41.6 (16.5)	41.4 (16.5)	42.6 (16.8)	
**Sex**				0.377
Male	72 (75.0%)	60 (76.9%)	12 (66.7%)	
Female	24 (25.0%)	18 (23.1%)	6 (33.3%)	
**BMI (kg/m2)**				0.950
Median (Q1, Q3)	23.5 (21.3, 26.2)	23.5 (21.3, 25.9)	23.8 (21.3, 26.6)	
Mean (SD)	23.8 (3.7)	23.7 (3.7)	23.9 (4.0)	
Missing	12	10	2	
**Charlson Comorbidity Index**				0.102
Median (Q1, Q3)	2.0 (0.5, 3.0)	2.0 (0.0, 3.0)	2.5 (2.0, 4.0)	
Mean (SD)	2.1 (1.9)	2.0 (2.0)	2.6 (1.7)	
**Diabetes mellitus**	24 (25.0%)	20 (25.6%)	4 (22.2%)	>0.999
**Chronic kidney disease**	43 (44.8%)	34 (43.6%)	9 (50.0%)	0.793
**Liver disease**	2 (2.1%)	2 (2.6%)	0 (0.0%)	>0.999
**Active neoplasm**	1 (1.0%)	1 (1.3%)	0 (0.0%)	>0.999
**Intravenous drug use**	3 (3.1%)	3 (3.8%)	0 (0.0%)	>0.999
**Previous endocarditis**	7 (7.3%)	5 (6.4%)	2 (11.1%)	0.612
**Prosthetic valve**	11 (11.5%)	9 (11.5%)	2 (11.1%)	>0.999
**CVC or Mahurkar catheter**	44 (45.8%)	36 (46.2%)	8 (44.4%)	>0.999
**Cardiac electronic device**	5 (5.3%)	5 (6.4%)	0 (0.0%)	0.581
Missing	1	0	1	
**Hemodialysis**	46 (47.9%)	37 (47.4%)	9 (50.0%)	>0.999
**Recent hospitalization (3 months)**	43 (44.8%)	34 (43.6%)	9 (50.0%)	0.793
**IE type**				0.865
Community-acquired	42 (44.7%)	33 (43.4%)	9 (50.0%)	
Healthcare-associated	50 (53.2%)	41 (53.9%)	9 (50.0%)	
Nosocomial	2 (2.1%)	2 (2.6%)	0 (0.0%)	
Missing	2	2	0	
**Prosthetic vs. native IE**				>0.999
Native	85 (88.5%)	69 (88.5%)	16 (88.9%)	
Prosthetic	11 (11.5%)	9 (11.5%)	2 (11.1%)	
**Affected side**				0.158
Left	48 (50.0%)	36 (46.2%)	12 (66.7%)	
Right	45 (46.9%)	40 (51.3%)	5 (27.8%)	
Bilateral	2 (2.1%)	1 (1.3%)	1 (5.6%)	
Unclassified	1 (1.0%)	1 (1.3%)	0 (0.0%)	
**Positive blood cultures**	65 (71.4%)	53 (70.7%)	12 (75.0%)	>0.999
Missing	5	3	2	
**Pathogen group**				0.627
Staphylococcus aureus	31 (32.3%)	26 (33.3%)	5 (27.8%)	
Streptococcus spp.	12 (12.5%)	9 (11.5%)	3 (16.7%)	
Enterococcus spp.	3 (3.1%)	2 (2.6%)	1 (5.6%)	
Coagulase-negative staphylococci	8 (8.3%)	6 (7.7%)	2 (11.1%)	
Gram-negative organisms	9 (9.4%)	9 (11.5%)	0 (0.0%)	
Fungi	4 (4.2%)	3 (3.8%)	1 (5.6%)	
Culture-negative	29 (30.2%)	23 (29.5%)	6 (33.3%)	
**LVEF (%)**				0.774

Continued on next page

Table S2 continued

Variable	Total (N=96)	No N = 78	Yes N = 18	p-value
Median (Q1, Q3)	60.0 (55.0, 63.0)	60.0 (54.0, 63.0)	59.0 (55.0, 64.0)	
Mean (SD)	57.5 (8.6)	57.3 (8.5)	58.3 (9.2)	
Missing	2	0	2	
**LVEF (category)**				>0.999
Preserved (≥50%)	80 (85.1%)	66 (84.6%)	14 (87.5%)	
Moderately reduced (40-49%)	10 (10.6%)	8 (10.3%)	2 (12.5%)	
Reduced (<40%)	4 (4.3%)	4 (5.1%)	0 (0.0%)	
Missing	2	0	2	
**Maximum vegetation dimension (mm)**				0.037
Median (Q1, Q3)	19.0 (13.0, 24.0)	18.0 (12.0, 23.0)	21.0 (19.0, 30.0)	
Mean (SD)	20.1 (9.6)	19.2 (9.8)	23.9 (8.3)	
Missing	15	12	3	
**Vegetation >10 mm**	69 (85.2%)	54 (81.8%)	15 (100.0%)	0.110
Missing	15	12	3	
**Embolism**	43 (44.8%)	34 (43.6%)	9 (50.0%)	0.793
**Acute heart failure**	26 (27.1%)	13 (16.7%)	13 (72.2%)	<0.001
**Vasopressor-requiring shock**	19 (19.8%)	8 (10.3%)	11 (61.1%)	<0.001
**Arrhythmias**	2 (2.1%)	2 (2.6%)	0 (0.0%)	>0.999
**Total complications**				<0.001
Median (Q1, Q3)	2.0 (1.0, 3.0)	2.0 (0.0, 3.0)	4.0 (3.0, 5.0)	
Mean (SD)	2.2 (1.6)	1.8 (1.5)	3.8 (1.2)	
**SOFA-2 score**				<0.001
Median (Q1, Q3)	3.0 (0.0, 4.0)	2.0 (0.0, 4.0)	4.0 (3.0, 6.0)	
Mean (SD)	2.7 (2.3)	2.3 (2.1)	4.5 (2.5)	
**RiskE score (points)**				0.009
Median (Q1, Q3)	9.0 (0.0, 13.0)	9.0 (0.0, 9.0)	14.0 (7.0, 21.0)	
Mean (SD)	8.9 (8.4)	7.5 (6.7)	15.2 (11.7)	
Missing	1	1	0	
**ICE score (points)**				0.093
Median (Q1, Q3)	7.0 (5.0, 9.0)	7.0 (5.0, 9.0)	9.0 (6.0, 10.0)	
Mean (SD)	7.3 (3.4)	7.0 (3.3)	8.6 (3.8)	
Missing	1	1	0	
**Surgical indication**	59 (74.7%)	42 (68.9%)	17 (94.4%)	0.032
Missing	17	17	0	
**Surgery performed**	60 (63.2%)	49 (63.6%)	11 (61.1%)	>0.999
Missing	1	1	0	
**Hospital stay (days)**				0.149
Median (Q1, Q3)	35.0 (25.0, 51.0)	31.0 (26.0, 47.0)	48.5 (19.0, 65.0)	
Mean (SD)	40.3 (25.3)	38.3 (23.2)	49.0 (32.1)	
Missing	1	1	0	
**In-hospital mortality**				0.005
Survivor	74 (77.1%)	65 (83.3%)	9 (50.0%)	
Death	22 (22.9%)	13 (16.7%)	9 (50.0%)	

[1]n (%)

[2]Wilcoxon rank sum test; Fisher's exact test

Table S3: Clinical Characteristics by Prolonged Hospital Stay (>69 Days, 90th Percentile)

Variable	Total (N=96)	No N = 87	Yes N = 9	p-value
**Age (years)**				0.191
Median (Q1, Q3)	37.5 (28.0, 52.5)	38.0 (27.0, 53.0)	32.0 (28.0, 43.0)	
Mean (SD)	41.6 (16.5)	42.4 (16.9)	34.2 (9.5)	
**Sex**				0.041
Male	72 (75.0%)	68 (78.2%)	4 (44.4%)	
Female	24 (25.0%)	19 (21.8%)	5 (55.6%)	
**BMI (kg/m2)**				0.789
Median (Q1, Q3)	23.5 (21.5, 26.2)	23.7 (21.5, 26.8)	23.4 (21.6, 25.4)	
Mean (SD)	23.8 (3.7)	23.8 (3.8)	23.1 (2.8)	
Missing	12	10	2	
**Charlson Comorbidity Index**				0.857
Median (Q1, Q3)	2.0 (0.0, 3.0)	2.0 (0.0, 3.0)	2.0 (2.0, 2.0)	
Mean (SD)	2.1 (2.0)	2.1 (2.0)	1.9 (1.1)	
**Diabetes mellitus**	24 (25.0%)	21 (24.1%)	3 (33.3%)	0.686
**Chronic kidney disease**	42 (43.8%)	36 (41.4%)	6 (66.7%)	0.173
**Liver disease**	2 (2.1%)	2 (2.3%)	0 (0.0%)	>0.999
**Active neoplasm**	1 (1.0%)	1 (1.1%)	0 (0.0%)	>0.999
**Intravenous drug use**	3 (3.1%)	3 (3.4%)	0 (0.0%)	>0.999
**Previous endocarditis**	7 (7.3%)	7 (8.0%)	0 (0.0%)	>0.999
**Prosthetic valve**	11 (11.5%)	11 (12.6%)	0 (0.0%)	0.592
**CVC or Mahurkar catheter**	43 (44.8%)	36 (41.4%)	7 (77.8%)	0.073
**Cardiac electronic device**	5 (5.3%)	5 (5.8%)	0 (0.0%)	>0.999
Missing	1	1	0	
**Hemodialysis**	45 (46.9%)	39 (44.8%)	6 (66.7%)	0.297
**Recent hospitalization (3 months)**	43 (44.8%)	37 (42.5%)	6 (66.7%)	0.291
**IE type**				0.323
Community-acquired	42 (45.2%)	40 (47.6%)	2 (22.2%)	
Healthcare-associated	49 (52.7%)	42 (50.0%)	7 (77.8%)	
Nosocomial	2 (2.2%)	2 (2.4%)	0 (0.0%)	
Missing	3	3	0	
**Prosthetic vs. native IE**				0.592
Native	85 (88.5%)	76 (87.4%)	9 (100.0%)	
Prosthetic	11 (11.5%)	11 (12.6%)	0 (0.0%)	
**Affected side**				0.484
Left	48 (50.5%)	45 (52.3%)	3 (33.3%)	
Right	44 (46.3%)	38 (44.2%)	6 (66.7%)	
Bilateral	2 (2.1%)	2 (2.3%)	0 (0.0%)	
Unclassified	1 (1.1%)	1 (1.2%)	0 (0.0%)	
Missing	1	1	0	
**Positive blood cultures**	65 (71.4%)	58 (70.7%)	7 (77.8%)	>0.999
Missing	5	5	0	
**Pathogen group**				0.040
Staphylococcus aureus	30 (31.3%)	28 (32.2%)	2 (22.2%)	
Streptococcus spp.	13 (13.5%)	13 (14.9%)	0 (0.0%)	
Enterococcus spp.	3 (3.1%)	3 (3.4%)	0 (0.0%)	
Coagulase-negative staphylococci	8 (8.3%)	6 (6.9%)	2 (22.2%)	
Gram-negative organisms	9 (9.4%)	6 (6.9%)	3 (33.3%)	
Fungi	4 (4.2%)	3 (3.4%)	1 (11.1%)	
Culture-negative	29 (30.2%)	28 (32.2%)	1 (11.1%)	
**LVEF (%)**				0.348

Continued on next page

Table S3 continued

Variable	Total (N=96)	No N = 87	Yes N = 9	p-value
Median (Q1, Q3)	60.0 (55.0, 63.0)	60.0 (54.0, 64.0)	57.0 (55.0, 59.0)	
Mean (SD)	57.5 (8.7)	57.5 (9.1)	57.3 (2.6)	
Missing	3	3	0	
**LVEF (category)**				0.729
Preserved (≥50%)	79 (84.9%)	70 (83.3%)	9 (100.0%)	
Moderately reduced (40-49%)	10 (10.8%)	10 (11.9%)	0 (0.0%)	
Reduced (<40%)	4 (4.3%)	4 (4.8%)	0 (0.0%)	
Missing	3	3	0	
**Maximum vegetation dimension (mm)**				0.302
Median (Q1, Q3)	19.0 (13.0, 24.0)	19.0 (13.0, 24.0)	23.0 (20.0, 24.0)	
Mean (SD)	20.2 (9.6)	20.1 (10.0)	21.1 (4.5)	
Missing	16	13	3	
**Vegetation >10 mm**	68 (85.0%)	62 (83.8%)	6 (100.0%)	0.584
Missing	16	13	3	
**Embolism**	43 (45.3%)	39 (45.3%)	4 (44.4%)	>0.999
Missing	1	1	0	
**Acute heart failure**	26 (27.4%)	22 (25.6%)	4 (44.4%)	0.251
Missing	1	1	0	
**Vasopressor-requiring shock**	19 (20.0%)	17 (19.8%)	2 (22.2%)	>0.999
Missing	1	1	0	
**Arrhythmias**	2 (2.1%)	2 (2.3%)	0 (0.0%)	>0.999
Missing	1	1	0	
**ICU admission**	18 (18.9%)	15 (17.4%)	3 (33.3%)	0.365
Missing	1	1	0	
**Total complications**				0.622
Median (Q1, Q3)	2.0 (1.0, 3.0)	2.0 (1.0, 3.0)	1.0 (0.0, 5.0)	
Mean (SD)	2.2 (1.6)	2.2 (1.5)	2.1 (2.5)	
Missing	1	1	0	
**SOFA-2 score**				0.017
Median (Q1, Q3)	3.0 (0.0, 4.0)	2.5 (0.0, 4.0)	4.0 (4.0, 5.0)	
Mean (SD)	2.7 (2.3)	2.5 (2.3)	4.2 (1.9)	
Missing	1	1	0	
**RiskE score (points)**				0.801
Median (Q1, Q3)	9.0 (0.0, 13.0)	9.0 (0.0, 13.0)	9.0 (0.0, 9.0)	
Mean (SD)	8.9 (8.4)	9.0 (8.5)	8.2 (7.8)	
Missing	2	2	0	
**ICE score (points)**				0.275
Median (Q1, Q3)	7.0 (5.0, 9.0)	8.0 (5.0, 9.0)	7.0 (5.0, 7.0)	
Mean (SD)	7.3 (3.4)	7.4 (3.5)	6.1 (2.3)	
Missing	2	2	0	
**Surgical indication**	59 (75.6%)	53 (75.7%)	6 (75.0%)	>0.999
Missing	18	17	1	
**Surgery performed**	60 (63.2%)	53 (61.6%)	7 (77.8%)	0.478
Missing	1	1	0	
**In-hospital mortality**				>0.999
Survivor	73 (76.8%)	66 (76.7%)	7 (77.8%)	
Death	22 (23.2%)	20 (23.3%)	2 (22.2%)	
Missing	1	1	0	

[1]n (%)

[2]Wilcoxon rank sum test; Fisher's exact test

Table S4: Clinical Characteristics by Complicated IE

Variable	Total (N=96)	No N = 37	Yes N = 59	p-value
**Age (years)**				0.039
Median (Q1, Q3)	37.5 (28.0, 52.5)	33.0 (27.0, 41.0)	46.0 (30.0, 54.0)	
Mean (SD)	41.6 (16.5)	37.8 (16.3)	44.1 (16.3)	
**Sex**				0.470
Male	72 (75.0%)	26 (70.3%)	46 (78.0%)	
Female	24 (25.0%)	11 (29.7%)	13 (22.0%)	
**BMI (kg/m2)**				0.518
Median (Q1, Q3)	23.5 (21.3, 26.2)	23.1 (21.6, 25.5)	23.7 (21.1, 27.1)	
Mean (SD)	23.8 (3.7)	23.4 (2.8)	24.0 (4.2)	
Missing	12	3	9	
**Charlson Comorbidity Index**				0.767
Median (Q1, Q3)	2.0 (0.5, 3.0)	2.0 (1.0, 2.0)	2.0 (0.0, 3.0)	
Mean (SD)	2.1 (1.9)	2.0 (1.8)	2.2 (2.0)	
**Diabetes mellitus**	24 (25.0%)	8 (21.6%)	16 (27.1%)	0.632
**Chronic kidney disease**	43 (44.8%)	21 (56.8%)	22 (37.3%)	0.091
**Liver disease**	2 (2.1%)	1 (2.7%)	1 (1.7%)	>0.999
**Active neoplasm**	1 (1.0%)	1 (2.7%)	0 (0.0%)	0.385
**Intravenous drug use**	3 (3.1%)	1 (2.7%)	2 (3.4%)	>0.999
**Previous endocarditis**	7 (7.3%)	2 (5.4%)	5 (8.5%)	0.703
**Prosthetic valve**	11 (11.5%)	3 (8.1%)	8 (13.6%)	0.522
**CVC or Mahurkar catheter**	44 (45.8%)	22 (59.5%)	22 (37.3%)	0.038
**Cardiac electronic device**	5 (5.3%)	4 (10.8%)	1 (1.7%)	0.074
Missing	1	0	1	
**Hemodialysis**	46 (47.9%)	23 (62.2%)	23 (39.0%)	0.036
**Recent hospitalization (3 months)**	43 (44.8%)	18 (48.6%)	25 (42.4%)	0.674
**IE type**				0.019
Community-acquired	42 (44.7%)	11 (29.7%)	31 (54.4%)	
Healthcare-associated	50 (53.2%)	26 (70.3%)	24 (42.1%)	
Nosocomial	2 (2.1%)	0 (0.0%)	2 (3.5%)	
Missing	2	0	2	
**Prosthetic vs. native IE**				0.522
Native	85 (88.5%)	34 (91.9%)	51 (86.4%)	
Prosthetic	11 (11.5%)	3 (8.1%)	8 (13.6%)	
**Affected side**				<0.001
Left	48 (50.0%)	8 (21.6%)	40 (67.8%)	
Right	45 (46.9%)	28 (75.7%)	17 (28.8%)	
Bilateral	2 (2.1%)	0 (0.0%)	2 (3.4%)	
Unclassified	1 (1.0%)	1 (2.7%)	0 (0.0%)	
**Positive blood cultures**	65 (71.4%)	27 (75.0%)	38 (69.1%)	0.638
Missing	5	1	4	
**Pathogen group**				0.408
Staphylococcus aureus	31 (32.3%)	13 (35.1%)	18 (30.5%)	
Streptococcus spp.	12 (12.5%)	4 (10.8%)	8 (13.6%)	
Enterococcus spp.	3 (3.1%)	0 (0.0%)	3 (5.1%)	
Coagulase-negative staphylococci	8 (8.3%)	4 (10.8%)	4 (6.8%)	
Gram-negative organisms	9 (9.4%)	6 (16.2%)	3 (5.1%)	
Fungi	4 (4.2%)	1 (2.7%)	3 (5.1%)	
Culture-negative	29 (30.2%)	9 (24.3%)	20 (33.9%)	
**LVEF (%)**				0.236

Continued on next page

Table S4 continued

Variable	Total (N=96)	No N = 37	Yes N = 59	p-value
Median (Q1, Q3)	60.0 (55.0, 63.0)	60.0 (55.0, 63.0)	58.0 (54.0, 63.0)	
Mean (SD)	57.5 (8.6)	58.6 (8.7)	56.8 (8.6)	
Missing	2	0	2	
**LVEF (category)**				0.053
Preserved (≥50%)	80 (85.1%)	33 (89.2%)	47 (82.5%)	
Moderately reduced (40-49%)	10 (10.6%)	1 (2.7%)	9 (15.8%)	
Reduced (<40%)	4 (4.3%)	3 (8.1%)	1 (1.8%)	
Missing	2	0	2	
**Maximum vegetation dimension (mm)**				0.664
Median (Q1, Q3)	19.0 (13.0, 24.0)	20.0 (12.0, 24.0)	19.0 (13.0, 22.5)	
Mean (SD)	20.1 (9.6)	21.4 (12.0)	19.3 (8.1)	
Missing	15	8	7	
**Vegetation >10 mm**	69 (85.2%)	23 (79.3%)	46 (88.5%)	0.333
Missing	15	8	7	
**Arrhythmias**	2 (2.1%)	0 (0.0%)	2 (3.4%)	0.521
**Total complications**				<0.001
Median (Q1, Q3)	2.0 (1.0, 3.0)	0.0 (0.0, 2.0)	3.0 (2.0, 4.0)	
Mean (SD)	2.2 (1.6)	0.8 (1.1)	3.0 (1.3)	
**SOFA-2 score**				0.270
Median (Q1, Q3)	3.0 (0.0, 4.0)	4.0 (0.0, 4.0)	3.0 (1.0, 4.0)	
Mean (SD)	2.7 (2.3)	2.3 (1.9)	2.9 (2.5)	
**RiskE score (points)**				0.039
Median (Q1, Q3)	9.0 (0.0, 13.0)	9.0 (0.0, 9.0)	9.0 (5.0, 14.0)	
Mean (SD)	8.9 (8.4)	6.2 (5.8)	10.6 (9.3)	
Missing	1	0	1	
**ICE score (points)**				0.305
Median (Q1, Q3)	7.0 (5.0, 9.0)	7.0 (5.0, 8.0)	7.5 (5.0, 9.0)	
Mean (SD)	7.3 (3.4)	6.7 (2.8)	7.7 (3.8)	
Missing	1	0	1	
**Surgical indication**	59 (74.7%)	13 (48.1%)	46 (88.5%)	<0.001
Missing	17	10	7	
**Surgery performed**	60 (63.2%)	23 (63.9%)	37 (62.7%)	>0.999
Missing	1	1	0	
**Hospital stay (days)**				0.447
Median (Q1, Q3)	35.0 (25.0, 51.0)	29.5 (23.5, 51.0)	37.0 (26.0, 50.0)	
Mean (SD)	40.3 (25.3)	40.4 (28.8)	40.3 (23.2)	
Missing	1	1	0	
**In-hospital mortality**				<0.001
Survivor	74 (77.1%)	37 (100.0%)	37 (62.7%)	
Death	22 (22.9%)	0 (0.0%)	22 (37.3%)	

[1]n (%)

[2]Wilcoxon rank sum test; Fisher's exact test

Table S5: Multivariable Logistic Regression Models for Secondary Outcomes

Outcome	Variable	GLM OR (95% CI)	*p*	Firth OR (95% CI)	*p*
ICU admission	Vasopressor-requiring shock	7.71 (2.06–28.91)	0.002	7.03 (1.99–25.90)	0.003
	Acute heart failure	7.81 (2.14–28.44)	0.002	7.05 (2.09–25.61)	0.002
Prolonged stay (>69 days, P90)	Female sex	3.35 (0.78–14.32)	0.103	3.20 (0.79–13.33)	0.100
	CVC or Mahurkar catheter	3.96 (0.75–20.93)	0.106	3.37 (0.80–19.18)	0.099
Complicated IE	Left-sided IE	7.25 (2.78–18.88)	<0.001	6.85 (2.78–18.37)	<0.001

Model performance (apparent AUC): ICU admission = 0.874; Prolonged stay = 0.738; Complicated IE = 0.725.

Table S6: Empirical Antimicrobial Regimens ($n = 95$)

Empirical regimen	Abbrev.	Type	n	%	Mortality
Ceftriaxone + Vancomycin	CRO+VA	Combination	35	36.8	6/35 (17.1%)
Ceftazidime + Vancomycin	CAZ+VA	Combination	8	8.4	1/8 (12.5%)
Ceftriaxone + Linezolid	CRO+LZD	Combination	5	5.3	1/5 (20%)
Ceftazidime	CAZ	Monotherapy	4	4.2	1/4 (25%)
Cefazolin	CEF	Monotherapy	3	3.2	2/3 (66.7%)
Ceftriaxone	CRO	Monotherapy	3	3.2	1/3 (33.3%)
Ceftriaxone + Clarithromycin	CRO+CLAR	Combination	3	3.2	1/3 (33.3%)
Meropenem	MEM	Monotherapy	3	3.2	2/3 (66.7%)
Piperacillin-tazobactam	TZP	Monotherapy	3	3.2	1/3 (33.3%)
Vancomycin + Amikacin	VA+AN	Combination	3	3.2	1/3 (33.3%)
Ceftazidime + Linezolid	CAZ+LZD	Combination	2	2.1	—
Cefepime + Vancomycin	FEP+VA	Combination	2	2.1	—
Meropenem + Linezolid	MEM+LZD	Combination	2	2.1	—
Meropenem + Vancomycin	MEM+VA	Combination	2	2.1	—
Ceftazidime + Amikacin	CAZ+AN	Combination	1	1.1	—
Ceftazidime + Ciprofloxacin	CAZ+CIP	Combination	1	1.1	—
Ceftazidime + Daptomycin	CAZ+DAP	Combination	1	1.1	—
Ceftazidime + Vancomycin + Ciprofloxacin	CAZ+VA+CIP	Combination	1	1.1	—
Ceftazidime + Vancomycin + Gentamicin	CAZ+VA+GEN	Combination	1	1.1	—
Cefazolin + Gentamicin	CEF+GEN	Combination	1	1.1	—
Cefazolin + Vancomycin + Amikacin	CEF+VA+AN	Combination	1	1.1	—
Ceftriaxone + Clindamycin	CRO+CLI	Combination	1	1.1	—
Ceftriaxone + Daptomycin	CRO+DAP	Combination	1	1.1	—
Ceftriaxone + Metronidazole	CRO+MTZ	Combination	1	1.1	—
Ceftriaxone + Vancomycin + TMP-SMX	CRO+VA+SXT	Combination	1	1.1	—
Daptomycin	DAP	Monotherapy	1	1.1	—
Daptomycin + Amikacin	DAP+AN	Combination	1	1.1	—
Cefepime	FEP	Monotherapy	1	1.1	—
Linezolid	LZD	Monotherapy	1	1.1	—
RIF + Linezolid	RIF+LZD	Combination	1	1.1	—
TMP-SMX	SXT	Monotherapy	1	1.1	—

Table S7: Extended Univariate Analysis — Crude Odds Ratios for In-Hospital Mortality

Block	Variable	Comparison	*n*	Ev.	Crude OR (95% CI)	*p*
Derived variables	S. aureus pathogen	S. aureus pathogen	58	15	0.22 (0.05-0.75)	0.021
	High-risk pathogen (Enterococcus/ Fungi/ CoNS)	High-risk pathogen	96	22	7.85 (2.43-27.24)	<0.001
	Culture-negative	Culture-negative	91	21	0.73 (0.22-2.14)	0.583
	Diagnostic delay (no initial IE suspicion)	Diagnostic delay (no initial IE suspicion)	96	22	0.26 (0.09-0.72)	0.010
	High comorbidity burden (Charlson $\geq$ 4)	High comorbidity burden (Charlson $\geq$ 4)	96	22	2.95 (1.00-8.59)	0.047
	Left-sided endocarditis	Left-sided endocarditis	96	22	6.60 (2.21-24.61)	0.002
	Structural complication (abscess/ perforation/ fistula/ aneurysm)	Structural complication	96	22	1.90 (0.73-5.12)	0.195
	Reduced LVEF (<50%)	Reduced LVEF (<50%)	96	22	0.52 (0.08-2.11)	0.413
Individual complications	Valvular perforation	Valvular perforation	96	22	1.55 (0.59-4.08)	0.368
	Valvular regurgitation	Valvular regurgitation	96	22	3.24 (1.08-12.07)	0.050
	Obstruction	Obstruction	96	22	NE	0.229
	Abscess	Abscess	96	22	0.96 (0.14-4.35)	0.958
	Aneurysm	Aneurysm	96	22	3.89 (0.85-17.95)	0.072
	Fistula	Fistula	96	22	1.71 (0.08-18.76)	0.666
	Prosthetic dehiscence	Prosthetic dehiscence	92	20	3.74 (0.14-97.48)	0.359
Clinical presentation	Fever	Fever	94	22	0.30 (0.01-7.69)	0.396
	Vascular or immunological phenomena	Vascular or immunological phenomena	94	21	2.52 (0.76-7.95)	0.118
	Heart murmur	Heart murmur	95	22	1.03 (0.39-2.69)	0.955
	Mobile vegetation	Mobile vegetation	90	19	0.40 (0.09-2.13)	0.246
Biomarkers	Initial lactate (mmol/L)	Per unit increase	40	11	1.00 (1.00-1.00)	0.441
	Procalcitonin (ng/mL)	Per unit increase	57	11	1.00 (NA-1.00)	0.316
Temporal variables	Days from symptoms to diagnosis	Per unit increase	95	22	1.01 (0.99-1.03)	0.205
	Days to antibiotic correction	Per unit increase	42	9	1.03 (0.86-1.20)	0.707
	Duration of bacteremia (days)	Per unit increase	52	12	1.13 (1.00-1.30)	0.051
Treatment and microbiology	Appropriate initial antibiotic therapy	Appropriate initial antibiotic therapy	55	12	0.26 (0.06-1.26)	0.085
	Antibiotic correction	Antibiotic correction	53	12	0.73 (0.17-3.83)	0.681
	Prior antibiotic use	Prior antibiotic use	96	22	2.95 (1.00-8.59)	0.047
	S. aureus colonization	Per unit increase	96	22	NE	1.000
Additional risk factors	Valvular heart disease	Valvular heart disease	95	22	0.79 (0.21-2.50)	0.707
	Congenital heart disease	Congenital heart disease	96	22	2.07 (0.72-5.77)	0.166
	Immunosuppression	Immunosuppression	96	22	0.53 (0.11-1.80)	0.350
	Recent infection (3 months)	Recent infection (3 months)	96	22	2.71 (0.81-8.68)	0.095
	Recent dental/surgical procedures	Recent dental/surgical procedures	96	22	0.54 (0.03-3.41)	0.578
	Initial IE diagnosis	Initial IE diagnosis	96	22	3.91 (1.39-11.12)	0.010

Continued on next page

Table S7 continued

Block	Variable	Comparison	n	Ev.	Crude OR (95% CI)	p
	Valvular compromise	Valvular compromise	96	22	12.78 (2.46-235.38)	0.015
Scores	qSOFA	Per unit increase	96	22	4.07 (1.88-9.87)	<0.001

OR: odds ratio; CI: confidence interval; NE: not estimable (zero-cell separation). Bold: $p < 0.05$.

Table S8: Multi-Model Comparison for In-Hospital Mortality Prediction

Panel A: Odds Ratios Across Model Specifications

Predictor	Model 1 OR (95% CI)	*p*	Model 2 OR (95% CI)	*p*	Firth OR (95% CI)	*p*
Age (per year)	1.05 (1.00-1.11)	0.049	—	—	—	—
Vasopressor-requiring shock	4.29 (0.91-22.98)	0.072	11.14 (2.67-55.52)	0.001	9.23 (2.40-40.61)	0.001
Left-sided IE	—	—	2.93 (0.62-15.92)	0.182	2.66 (0.62-12.68)	0.185
Acute heart failure	45.58 (8.42-462.31)	<0.001	12.11 (3.12-55.72)	<0.001	10.01 (2.78-41.07)	<0.001
Surgery	0.28 (0.04-1.60)	0.173	—	—	—	—

Panel B: Performance Metrics

Metric	Model 1	Model 2	RiskE	ICE
N (events)	95 (22)	96 (22)	95	95
Predictors	4	3	—	—
EPV	5.5	7.3	—	—
AUC (95% CI)	0.933 (0.881-0.986)	0.922 (0.869-0.975)	0.597	0.632
Corrected AUC	0.916	0.908	—	—
AIC	62	64.8	—	—
Hosmer-Lemeshow p	0.961	0.505	—	—
Brier score	0.0861	0.0923	—	—
Nagelkerke R2	0.626	0.583	—	—

Panel C: DeLong Pairwise Comparisons

Comparison	AUC Diff	Z (DeLong)	p
Model 2 vs Model 1	-0.011	-0.86	0.3876
Model 2 vs RiskE	0.334	4.69	<0.0001
Model 2 vs ICE	0.295	4.08	<0.0001
Model 1 vs RiskE	0.345	5.10	<0.0001
Model 1 vs ICE	0.307	4.52	<0.0001

Table S9: Combined global WISCA: non-Bayesian and Bayesian empirical coverage estimates for 54 antimicrobial regimens in infective endocarditis

		Non-Bayesian WISCA		Bayesian WISCA		
Regimen	Type	Cov %	95% CI	Median %	95% HDI	Δ (pp)
VAN+GEN	Combo	0.71	(0.59–0.81)	0.87	(0.83–0.91)	+16.4
MEM+GEN	Combo	0.72	(0.60–0.82)	0.87	(0.83–0.91)	+14.9
MEM+VAN	Combo	0.91	(0.84–0.97)	0.87	(0.84–0.90)	-4.3
MEM+LZD	Combo	0.91	(0.84–0.97)	0.87	(0.84–0.90)	-4.3
MEM+DAP	Combo	0.91	(0.84–0.97)	0.87	(0.84–0.90)	-4.3
TGC	Mono	0.72	(0.54–0.81)	0.87	(0.80–0.93)	+14.8
FEP+LZD	Combo	0.90	(0.82–0.96)	0.86	(0.83–0.90)	-3.2
FEP+VAN	Combo	0.90	(0.82–0.96)	0.86	(0.83–0.90)	-3.3
FEP+DAP	Combo	0.90	(0.82–0.96)	0.86	(0.83–0.89)	-3.3
CRO+GEN	Combo	0.71	(0.59–0.81)	0.85	(0.81–0.90)	+14.7
CRO+LZD	Combo	0.87	(0.78–0.94)	0.85	(0.82–0.89)	-1.6
SAM+GEN	Combo	0.65	(0.51–0.76)	0.85	(0.81–0.89)	+20.5
CRO+DAP	Combo	0.87	(0.78–0.94)	0.85	(0.82–0.89)	-1.6
CRO+VAN	Combo	0.87	(0.78–0.94)	0.85	(0.82–0.89)	-1.6
CFZ+GEN	Combo	0.66	(0.50–0.76)	0.85	(0.80–0.89)	+18.9
CFZ+VAN	Combo	0.74	(0.59–0.82)	0.85	(0.81–0.89)	+11.5
CFZ+LZD	Combo	0.74	(0.59–0.82)	0.85	(0.81–0.89)	+11.5
CFZ+DAP	Combo	0.74	(0.59–0.82)	0.85	(0.81–0.89)	+11.4
AMP+GEN	Combo	0.62	(0.49–0.74)	0.85	(0.80–0.89)	+23.2
VAN+AN	Combo	0.72	(0.60–0.82)	0.85	(0.81–0.89)	+12.7
MEM+AN	Combo	0.72	(0.60–0.82)	0.85	(0.81–0.89)	+12.6
ETP+VAN	Combo	0.82	(0.74–0.91)	0.85	(0.81–0.88)	+2.3
ETP+DAP	Combo	0.82	(0.74–0.91)	0.85	(0.81–0.88)	+2.3
ETP+LZD	Combo	0.82	(0.74–0.91)	0.85	(0.81–0.88)	+2.3
CAZ+GEN	Combo	0.71	(0.57–0.81)	0.83	(0.78–0.88)	+12.8
CAZ+LZD	Combo	0.90	(0.82–0.96)	0.83	(0.79–0.87)	-6.4
CAZ+VAN	Combo	0.90	(0.82–0.96)	0.83	(0.79–0.87)	-6.4
CAZ+DAP	Combo	0.90	(0.82–0.96)	0.83	(0.79–0.87)	-6.4
CRO+AN	Combo	0.72	(0.60–0.82)	0.82	(0.78–0.87)	+10.2
SAM+AN	Combo	0.68	(0.54–0.78)	0.82	(0.78–0.86)	+14.5
DOX	Mono	0.60	(0.44–0.71)	0.82	(0.73–0.90)	+22.4
CFZ+AN	Combo	0.66	(0.50–0.76)	0.82	(0.77–0.86)	+15.7
AMP+AN	Combo	0.65	(0.51–0.75)	0.82	(0.77–0.86)	+17.0
PEN+GEN	Combo	0.62	(0.49–0.74)	0.81	(0.75–0.88)	+19.6
GEN	Mono	0.69	(0.57–0.79)	0.81	(0.73–0.87)	+11.6

Continued on next page

Table S9 (continued)

		Non-Bayesian WISCA		Bayesian WISCA		
Regimen	Type	Cov %	95% CI	Median %	95% HDI	Δ (pp)
LZD	Mono	0.81	(0.72–0.90)	0.81	(0.75–0.86)	-0.3
VAN	Mono	0.81	(0.72–0.90)	0.81	(0.75–0.86)	-0.3
CPT	Mono	0.81	(0.72–0.90)	0.81	(0.75–0.86)	-0.3
DAP	Mono	0.81	(0.72–0.90)	0.81	(0.75–0.86)	-0.3
IPM	Mono	0.81	(0.72–0.90)	0.81	(0.75–0.86)	-0.3
MEM	Mono	0.81	(0.72–0.90)	0.81	(0.75–0.86)	-0.3
FEP	Mono	0.79	(0.71–0.88)	0.79	(0.74–0.85)	-0.0
CAZ+AN	Combo	0.71	(0.57–0.81)	0.79	(0.75–0.84)	+8.7
CTX	Mono	0.75	(0.65–0.84)	0.76	(0.70–0.81)	+0.7
CRO	Mono	0.75	(0.65–0.84)	0.76	(0.70–0.81)	+0.7
PEN+AN	Combo	0.60	(0.47–0.71)	0.75	(0.68–0.82)	+15.0
SAM	Mono	0.72	(0.60–0.82)	0.75	(0.68–0.81)	+3.2
CFZ	Mono	0.63	(0.49–0.74)	0.75	(0.67–0.82)	+11.6
AMP	Mono	0.69	(0.56–0.79)	0.74	(0.67–0.81)	+5.1
AN	Mono	0.63	(0.51–0.75)	0.74	(0.65–0.82)	+10.6
ETP	Mono	0.68	(0.57–0.78)	0.74	(0.67–0.80)	+6.1
LVX	Mono	0.54	(0.37–0.66)	0.71	(0.63–0.79)	+17.5
CAZ	Mono	0.65	(0.54–0.76)	0.67	(0.60–0.74)	+1.9
PEN	Mono	0.29	(0.18–0.41)	0.35	(0.25–0.45)	+6.0

WISCA: Weighted-Incidence Syndromic Combination Antibiogram. Species-level analysis (23 species, 68 isolates, 54 regimens). Non-Bayesian: coverage = Σ(prevalence × susceptibility); 95% CI by bootstrap (B = 2000). Bayesian: hierarchical logistic model (brms/Stan); random intercepts for species and antibiotic; posterior median and 95% HDI from 8000 draws (4 chains × 2000 post-warmup). Δ = Bayesian median − non-Bayesian coverage. Combo susceptibility: OR logic (effective if $\geq$ 1 drug susceptible). Fungi (n = 4) included with susceptibility = 0 for all antibacterials.

Abbreviations: VAN = vancomycin, GEN = gentamicin, MEM = meropenem, LZD = linezolid, DAP = daptomycin, TGC = tigecycline, FEP = cefepime, CRO = ceftriaxone, CAZ = ceftazidime, CTX = cefotaxime, ETP = ertapenem, IPM = imipenem, CPT = ceftaroline, SAM = ampicillin/sulbactam, AMP = ampicillin, CFZ = cefazolin/cephalothin, PEN = penicillin G, AN = amikacin, DOX = doxycycline, LVX = levofloxacin.

Table S10: Stratified WISCA by IE type: non-Bayesian and Bayesian empirical coverage estimates (community-acquired vs. healthcare-associated infective endocarditis)

		Non-Bayesian WISCA		Bayesian WISCA		
Regimen	Type	Community	Healthcare	Community	Healthcare	Δ (pp)
VAN+GEN	Combo	0.36 (0.20–0.56)	0.71 (0.56–0.83)	0.91 (0.87–0.95)	0.84 (0.79–0.88)	+7.6
MEM+GEN	Combo	0.36 (0.20–0.56)	0.73 (0.59–0.85)	0.91 (0.87–0.95)	0.84 (0.79–0.88)	+7.5
TGC	Mono	0.56 (0.28–0.76)	0.80 (0.61–0.90)	0.91 (0.85–0.96)	0.84 (0.76–0.91)	+7.4
MEM+LZD	Combo	0.96 (0.88–1.00)	0.88 (0.78–0.98)	0.91 (0.88–0.94)	0.83 (0.79–0.87)	+7.6
MEM+VAN	Combo	0.96 (0.88–1.00)	0.88 (0.78–0.98)	0.91 (0.88–0.94)	0.83 (0.79–0.87)	+7.6
MEM+DAP	Combo	0.96 (0.84–1.00)	0.88 (0.78–0.98)	0.91 (0.88–0.94)	0.83 (0.79–0.87)	+7.6
FEP+VAN	Combo	0.96 (0.88–1.00)	0.85 (0.73–0.95)	0.91 (0.87–0.94)	0.83 (0.79–0.87)	+7.7
FEP+DAP	Combo	0.96 (0.84–1.00)	0.85 (0.73–0.95)	0.91 (0.87–0.94)	0.83 (0.79–0.87)	+7.7
FEP+LZD	Combo	0.96 (0.88–1.00)	0.85 (0.73–0.95)	0.91 (0.87–0.94)	0.83 (0.79–0.87)	+7.7
VAN+AN	Combo	0.36 (0.20–0.56)	0.73 (0.59–0.85)	0.90 (0.86–0.93)	0.82 (0.77–0.86)	+8.0
MEM+AN	Combo	0.36 (0.20–0.56)	0.73 (0.59–0.85)	0.90 (0.86–0.94)	0.82 (0.77–0.86)	+8.1
CRO+GEN	Combo	0.36 (0.20–0.56)	0.71 (0.56–0.83)	0.90 (0.85–0.94)	0.82 (0.76–0.87)	+8.1
CRO+VAN	Combo	0.92 (0.80–1.00)	0.83 (0.71–0.93)	0.90 (0.86–0.93)	0.82 (0.77–0.86)	+8.1
CRO+LZD	Combo	0.92 (0.80–1.00)	0.83 (0.71–0.93)	0.90 (0.86–0.93)	0.81 (0.77–0.86)	+8.1
CRO+DAP	Combo	0.92 (0.80–1.00)	0.83 (0.71–0.93)	0.90 (0.86–0.93)	0.81 (0.77–0.86)	+8.1
SAM+GEN	Combo	0.32 (0.16–0.52)	0.63 (0.49–0.78)	0.90 (0.85–0.94)	0.81 (0.76–0.87)	+8.1
CFZ+GEN	Combo	0.28 (0.08–0.48)	0.66 (0.49–0.80)	0.89 (0.85–0.94)	0.81 (0.76–0.87)	+8.1
CFZ+VAN	Combo	0.68 (0.44–0.84)	0.73 (0.59–0.85)	0.89 (0.86–0.93)	0.81 (0.77–0.86)	+8.2
CFZ+LZD	Combo	0.68 (0.44–0.84)	0.73 (0.59–0.85)	0.89 (0.86–0.93)	0.81 (0.77–0.86)	+8.2
CFZ+DAP	Combo	0.68 (0.44–0.84)	0.73 (0.59–0.85)	0.89 (0.86–0.93)	0.81 (0.77–0.86)	+8.1
AMP+GEN	Combo	0.32 (0.16–0.52)	0.59 (0.44–0.73)	0.89 (0.85–0.94)	0.81 (0.76–0.87)	+8.2
ETP+VAN	Combo	0.80 (0.60–0.92)	0.83 (0.71–0.93)	0.89 (0.86–0.93)	0.81 (0.77–0.85)	+8.3
ETP+DAP	Combo	0.80 (0.60–0.92)	0.83 (0.71–0.93)	0.89 (0.86–0.93)	0.81 (0.76–0.85)	+8.3
ETP+LZD	Combo	0.80 (0.60–0.92)	0.83 (0.71–0.93)	0.89 (0.85–0.93)	0.81 (0.76–0.85)	+8.2
CAZ+GEN	Combo	0.36 (0.16–0.56)	0.71 (0.56–0.83)	0.88 (0.83–0.93)	0.79 (0.74–0.86)	+8.6
CAZ+VAN	Combo	0.96 (0.84–1.00)	0.85 (0.73–0.95)	0.88 (0.84–0.92)	0.79 (0.75–0.84)	+8.6
CAZ+LZD	Combo	0.96 (0.84–1.00)	0.85 (0.73–0.95)	0.88 (0.84–0.92)	0.79 (0.74–0.84)	+8.6
CAZ+DAP	Combo	0.96 (0.80–1.00)	0.85 (0.73–0.95)	0.88 (0.84–0.92)	0.79 (0.75–0.84)	+8.6
CRO+AN	Combo	0.36 (0.20–0.56)	0.73 (0.59–0.85)	0.88 (0.84–0.92)	0.79 (0.74–0.84)	+8.7
SAM+AN	Combo	0.32 (0.16–0.52)	0.68 (0.51–0.83)	0.88 (0.83–0.92)	0.79 (0.73–0.84)	+8.7
CFZ+AN	Combo	0.28 (0.08–0.48)	0.66 (0.49–0.80)	0.88 (0.83–0.92)	0.79 (0.73–0.84)	+8.8
AMP+AN	Combo	0.32 (0.16–0.52)	0.63 (0.46–0.78)	0.87 (0.83–0.92)	0.78 (0.73–0.84)	+8.8
DOX	Mono	0.44 (0.20–0.64)	0.68 (0.51–0.80)	0.87 (0.80–0.94)	0.78 (0.69–0.87)	+8.7
PEN+GEN	Combo	0.32 (0.16–0.52)	0.59 (0.44–0.73)	0.86 (0.80–0.92)	0.77 (0.70–0.85)	+9.0
GEN	Mono	0.32 (0.16–0.52)	0.71 (0.56–0.83)	0.86 (0.79–0.92)	0.77 (0.68–0.85)	+9.1

Continued on next page

Table S10 (continued)

		Non-Bayesian WISCA		Bayesian WISCA		
Regimen	Type	Community	Healthcare	Community	Healthcare	Δ (pp)
VAN	Mono	0.92 (0.80–1.00)	0.73 (0.59–0.85)	0.86 (0.80–0.90)	0.77 (0.70–0.82)	+9.1
LZD	Mono	0.92 (0.80–1.00)	0.73 (0.59–0.85)	0.86 (0.81–0.91)	0.76 (0.70–0.83)	+9.2
CPT	Mono	0.92 (0.80–1.00)	0.73 (0.59–0.85)	0.86 (0.80–0.90)	0.76 (0.70–0.83)	+9.1
DAP	Mono	0.92 (0.80–1.00)	0.73 (0.59–0.85)	0.86 (0.80–0.90)	0.76 (0.70–0.83)	+9.1
MEM	Mono	0.84 (0.68–0.96)	0.78 (0.66–0.90)	0.86 (0.80–0.90)	0.76 (0.70–0.82)	+9.1
IPM	Mono	0.84 (0.68–0.96)	0.78 (0.66–0.90)	0.86 (0.80–0.90)	0.76 (0.70–0.82)	+9.1
CAZ+AN	Combo	0.32 (0.12–0.52)	0.73 (0.59–0.85)	0.85 (0.81–0.90)	0.76 (0.71–0.82)	+9.3
FEP	Mono	0.84 (0.68–0.96)	0.76 (0.61–0.88)	0.84 (0.79–0.89)	0.75 (0.69–0.81)	+9.4
PEN+AN	Combo	0.24 (0.08–0.40)	0.63 (0.46–0.78)	0.83 (0.76–0.89)	0.73 (0.65–0.80)	+10.0
AN	Mono	0.20 (0.08–0.36)	0.73 (0.59–0.85)	0.82 (0.74–0.89)	0.71 (0.62–0.80)	+10.2
CTX	Mono	0.80 (0.64–0.96)	0.71 (0.56–0.83)	0.81 (0.75–0.86)	0.71 (0.64–0.77)	+10.2
CRO	Mono	0.80 (0.64–0.96)	0.71 (0.56–0.83)	0.81 (0.75–0.86)	0.71 (0.64–0.77)	+10.2
SAM	Mono	0.80 (0.60–0.92)	0.63 (0.49–0.78)	0.81 (0.74–0.87)	0.70 (0.63–0.78)	+10.3
CFZ	Mono	0.56 (0.32–0.76)	0.63 (0.46–0.78)	0.80 (0.73–0.87)	0.70 (0.62–0.78)	+10.4
AMP	Mono	0.80 (0.60–0.92)	0.59 (0.44–0.73)	0.80 (0.73–0.86)	0.69 (0.61–0.77)	+10.5
ETP	Mono	0.60 (0.36–0.76)	0.71 (0.56–0.85)	0.79 (0.73–0.85)	0.69 (0.61–0.76)	+10.6
LVX	Mono	0.56 (0.20–0.72)	0.51 (0.33–0.68)	0.77 (0.69–0.85)	0.66 (0.57–0.74)	+11.0
CAZ	Mono	0.72 (0.52–0.88)	0.61 (0.46–0.76)	0.74 (0.66–0.80)	0.62 (0.54–0.69)	+11.6
PEN	Mono	0.32 (0.16–0.52)	0.27 (0.13–0.41)	0.46 (0.35–0.57)	0.28 (0.17–0.39)	+18.2

WISCA: Weighted-Incidence Syndromic Combination Antibiogram. Species-level analysis. Community: n = 25 isolates; Healthcare-associated: n = 41 isolates. Non-Bayesian: coverage = Σ(prevalence × susceptibility); 95% CI by bootstrap (B = 2000). Bayesian: hierarchical logistic model (brms/Stan) with IE type as fixed effect; random intercepts for species and antibiotic; posterior median and 95% HDI. Δ = Bayesian community − healthcare (pp). Combo susceptibility: OR logic. Fungi included (susceptibility = 0).

Abbreviations: VAN = vancomycin, GEN = gentamicin, MEM = meropenem, LZD = linezolid, DAP = daptomycin, TGC = tigecycline, FEP = cefepime, CRO = ceftriaxone, CAZ = ceftazidime, CTX = cefotaxime, ETP = ertapenem, IPM = imipenem, CPT = ceftaroline, SAM = ampicillin/sulbactam, AMP = ampicillin, CFZ = cefazolin/cephalothin, PEN = penicillin G, AN = amikacin, DOX = doxycycline, LVX = levofloxacin.

Table S11: Convergence diagnostics for Bayesian WISCA models

Global model: 49 parameters. Stratified model: 49 parameters. All R-hat < 1.01; zero divergent transitions. MCMC: 4 chains x 4,000 iterations (2,000 warmup).

Model	Parameter	$\hat{R}$	ESS (bulk)	ESS (tail)
**Global**	**SUMMARY**	**1.0052**	**1,080**	**2,139**
**Stratified**	**SUMMARY**	**1.0053**	**1,076**	**2,176**
Global	Species: Staphylococcus aureus	1.0052	1,080	2,139
Global	Intercept	1.0045	1,086	2,357
Global	Species: Enterococcus faecalis	1.0044	1,195	2,513
Global	Species: Pseudomonas aeruginosa	1.0043	1,278	2,412
Global	Species: Klebsiella pneumoniae	1.0042	1,207	2,695
Global	Species: Staphylococcus epidermidis	1.0040	1,136	2,311
Global	Intercept	1.0037	1,128	2,321
Global	Species: Staphylococcus coagulase-negative	1.0037	1,243	2,565
Global	Species: Streptococcus gordonii	1.0036	1,860	2,985
Global	Species: Streptococcus thoraltensis	1.0035	1,674	3,552
Global	Species: Escherichia coli	1.0034	1,190	2,373
Global	Species: Staphylococcus haemolyticus	1.0034	1,259	2,645
Global	Species: Stenotrophomonas maltophilia	1.0032	1,454	2,952
Global	Species: Enterobacter cloacae	1.0031	1,416	2,936
Global	Species: Streptococcus mitis	1.0030	1,507	2,792
Global	Species: Streptococcus sanguinis	1.0027	2,386	3,613
Global	Species: Staphylococcus saprophyticus	1.0021	2,549	3,938
Global	Antibiotic: Vancomycin	1.0014	5,350	5,155
Global	Species: Streptococcus agalactiae	1.0014	4,649	4,760
Global	Age (scaled)	1.0012	9,121	5,833
Global	Antibiotic: Cefotaxime	1.0012	4,745	5,431
Global	Species: Peptostreptococcus species	1.0010	5,250	5,267
Global	Antibiotic: Tigecycline	1.0009	9,120	5,265
Global	Antibiotic: Ceftriaxone	1.0008	5,113	5,289
Global	Species: Streptococcus pneumoniae	1.0008	4,794	5,074
Global	Sex	1.0007	9,111	5,613
Global	Antibiotic: Meropenem	1.0007	5,536	5,406
Global	Antibiotic: Ampicillin	1.0006	6,048	5,832
Global	Antibiotic: Ceftaroline	1.0006	5,551	5,127
Global	Antibiotic: Doxycycline	1.0006	8,481	5,649
Global	Antibiotic: Penicillin G	1.0006	4,083	5,511
Global	SD (species)	1.0005	2,166	3,651
Global	Antibiotic: Ceftazidime	1.0005	4,874	5,342
Global	Antibiotic: Daptomycin	1.0005	5,460	5,393
Global	Antibiotic: Ertapenem	1.0005	5,496	4,894
Global	Antibiotic: Linezolid	1.0005	5,918	5,664
Global	Species: Candida albicans	1.0005	4,032	4,951
Global	Species: Streptococcus gallolyticus subspecies pasteurianus	1.0004	5,602	5,214
Global	SD (antibiotic)	1.0003	2,823	4,335
Global	Antibiotic: Cefepime	1.0003	5,798	5,504
Global	Antibiotic: Imipenem	1.0003	5,817	5,887
Global	Species: Candida albicans, Candida glabrata	1.0003	4,539	4,589
Global	Species: Candida tropicalis	1.0003	4,732	3,977

Continued on next page

Table S11 (continued)

Model	Parameter	$\hat{R}$	ESS (bulk)	ESS (tail)
Global	Species: Cryptococcus neoformans	1.0003	4,591	4,776
Global	Antibiotic: Amikacin	1.0002	7,006	6,069
Global	Antibiotic: Cefazolin	1.0001	6,545	5,884
Global	Antibiotic: Gentamicin	1.0000	6,619	5,404
Global	Antibiotic: Levofloxacin	1.0000	6,322	5,619
Global	Antibiotic: Ampicillin/sulbactam	1.0000	6,023	5,993
Stratified	Species: Staphylococcus epidermidis	1.0053	1,124	2,403
Stratified	Species: Escherichia coli	1.0048	1,128	2,535
Stratified	Species: Klebsiella pneumoniae	1.0045	1,229	2,625
Stratified	Species: Pseudomonas aeruginosa	1.0045	1,159	2,523
Stratified	Species: Staphylococcus aureus	1.0045	1,077	2,396
Stratified	Species: Staphylococcus coagulase-negative	1.0044	1,156	2,881
Stratified	Intercept	1.0042	1,076	2,184
Stratified	Species: Stenotrophomonas maltophilia	1.0042	1,372	2,592
Stratified	Species: Staphylococcus haemolyticus	1.0041	1,205	2,710
Stratified	Species: Streptococcus mitis	1.0040	1,415	3,123
Stratified	Species: Streptococcus gordonii	1.0038	1,695	3,452
Stratified	Intercept	1.0036	1,149	2,508
Stratified	Species: Enterococcus faecalis	1.0036	1,168	2,176
Stratified	Species: Enterobacter cloacae	1.0035	1,411	2,604
Stratified	Species: Streptococcus thoraltensis	1.0023	1,660	3,146
Stratified	Antibiotic: Imipenem	1.0011	4,739	5,367
Stratified	Species: Candida albicans, Candida glabrata	1.0010	3,950	4,549
Stratified	Species: Candida albicans	1.0009	4,128	4,864
Stratified	Species: Streptococcus agalactiae	1.0009	5,085	4,358
Stratified	Species: Streptococcus gallolyticus subspecies pasteurianus	1.0009	4,703	4,595
Stratified	Antibiotic: Daptomycin	1.0008	4,726	5,340
Stratified	Antibiotic: Tigecycline	1.0008	7,637	5,809
Stratified	Species: Candida tropicalis	1.0008	4,774	4,856
Stratified	Species: Cryptococcus neoformans	1.0008	3,860	4,925
Stratified	Species: Streptococcus sanguinis	1.0008	4,658	4,718
Stratified	Antibiotic: Amikacin	1.0007	6,526	5,871
Stratified	Antibiotic: Vancomycin	1.0007	4,208	5,627
Stratified	Antibiotic: Cefepime	1.0006	4,652	5,727
Stratified	Antibiotic: Gentamicin	1.0006	6,714	5,979
Stratified	Antibiotic: Levofloxacin	1.0006	5,258	5,560
Stratified	Sex	1.0005	10,229	6,179
Stratified	Antibiotic: Ertapenem	1.0005	4,666	5,479
Stratified	Antibiotic: Ampicillin/sulbactam	1.0005	4,634	5,482
Stratified	Antibiotic: Ceftriaxone	1.0004	4,348	5,080
Stratified	Antibiotic: Linezolid	1.0004	4,772	5,052
Stratified	Antibiotic: Ceftaroline	1.0003	4,664	5,754
Stratified	Antibiotic: Cefotaxime	1.0003	4,395	5,541
Stratified	Antibiotic: Penicillin G	1.0003	3,674	5,437
Stratified	Species: Streptococcus pneumoniae	1.0003	4,732	5,369
Stratified	Age (scaled)	1.0002	8,669	6,741
Stratified	Antibiotic: Ampicillin	1.0002	5,014	5,265
Stratified	Antibiotic: Ceftazidime	1.0002	3,942	5,610
Stratified	Antibiotic: Cefazolin	1.0002	5,432	5,296

Continued on next page

Table S11 (continued)

Model	Parameter	$\hat{R}$	ESS (bulk)	ESS (tail)
Stratified	Antibiotic: Doxycycline	1.0002	7,594	6,278
Stratified	Species: Peptostreptococcus species	1.0002	5,451	5,371
Stratified	IE type	1.0001	8,408	5,824
Stratified	SD (antibiotic)	1.0001	2,650	4,016
Stratified	Antibiotic: Meropenem	1.0000	4,871	5,364
Stratified	SD (species)	0.9999	2,430	3,843
